# A multi-omics investigation of the molecular characteristics and classification of six metabolic syndrome relevant diseases

**DOI:** 10.7150/thno.41106

**Published:** 2020-01-12

**Authors:** Di Chen, Xinjie Zhao, Zhigang Sui, Huan Niu, Luonan Chen, Cheng Hu, Qiuhui Xuan, Xuhong Hou, Rong Zhang, Lina Zhou, Yanli Li, Huiming Yuan, Yukui Zhang, Jiarui Wu, Lihua Zhang, Ren'an Wu, Hai-Long Piao, Guowang Xu, Weiping Jia

**Affiliations:** 1 CAS Key Laboratory of Separation Science for Analytical Chemistry, Dalian Institute of Chemical Physics, Chinese Academy of Sciences, Dalian 116023, China.; 2 Laboratory of High-Resolution Mass Spectrometry Technologies, Dalian Institute of Chemical Physics, Chinese Academy of Sciences (CAS), Dalian 116023, China; 3 The University of Chinese Academy of Sciences, Beijing 100049, China; 4 Key Laboratory of Systems Biology, CAS Center for Excellence in Molecular Cell Science, Institute of Biochemistry and Cell Biology, University of Chinese Academy of Sciences, Chinese Academy of Sciences, 320 Yue-Yang Road, Shanghai 200031, China; 5 Shanghai Diabetes Institute, Shanghai Key Laboratory of Diabetes Mellitus, Shanghai Key Clinical Center for Metabolic Diseases, Shanghai Jiao Tong University Affiliated Sixth People's Hospital, 600 Yishan Road, Shanghai, 200233, People's Republic of China

**Keywords:** metabolic syndrome, metabolic diseases, multi-omics data, lipid and glucose metabolism, disease subtype identification

## Abstract

Metabolic syndrome (MTS) is a cluster of concurrent metabolic abnormal conditions. MTS and its component metabolic diseases are heterogeneous and closely related, making their relationships complicated, thus hindering precision treatment.

**Methods**: We collected seven groups of samples (group a: healthy individuals; group b: obesity; group c: MTS; group d: hyperglycemia, group e: hypertension, group f: hyperlipidemia; group g: type II diabetes, n=7 for each group). We examined the molecular characteristics of each sample by metabolomic, proteomic and peptidomic profiling analysis. The differential molecules (including metabolites, proteins and peptides) between each disease group and the healthy group were recognized by statistical analyses. Furthermore, a two-step clustering workflow which combines multi-omics and clinical information was used to redefine molecularly and clinically differential groups. Meanwhile, molecular, clinical, network and pathway based analyses were used to identify the group-specific biological features.

**Results**: Both shared and disease-specific molecular profiles among the six types of diseases were identified. Meanwhile, the patients were stratified into three distinct groups which were different from original disease definitions but presented significant differences in glucose and lipid metabolism (Group 1: relatively favorable metabolic conditions; Group 2: severe dyslipidemia; Group 3: dysregulated insulin and glucose). Group specific biological signatures were also systematically described. The dyslipidemia group showed higher levels in multiple lipid metabolites like phosphatidylserine and phosphatidylcholine, and showed significant up-regulations in lipid and amino acid metabolism pathways. The glucose dysregulated group showed higher levels in many polypeptides from proteins contributing to immune response. The another group, with better glucose/lipid metabolism ability, showed higher levels in lipid regulating enzymes like the lecithin cholesterol acyltransferase and proteins involved in complement and coagulation cascades.

**Conclusions**: This multi-omics based study provides a general view of the complex relationships and an alternative classification for various metabolic diseases where the cross-talk or compensatory mechanism between the immune and metabolism systems plays a critical role.

## Introduction

Metabolic syndrome (MTS) refers to a cluster of abnormal metabolic conditions such as hyperglycemia, obesity and hyperlipidemia that occur together 1, 2. It will increase the risk of various diseases 3, especially type 2 diabetes (T2D) 4 and cardiovascular disease 5. MTS is increasingly common, and up to one-third of U.S. adults suffer from it 6. Early diagnosis and treatment of MTS can greatly improve people's health.

MTS is closely related with dys-regulated glucose and lipid metabolisms. The abnormal glucose metabolism is linked to insulin resistance. Under the insulin resistance conditions, the cells cannot respond normally to insulin, and glucose from the bloodstream cannot enter the cells as easily. The lipid metabolism is central to energy generation. Abnormal lipid metabolism can predict future overweight, MTS and diabetes 7. Glucose and lipid metabolisms are highly correlated, and lipid changes can be both the cause and consequence of impaired glucose metabolism 8. Correspondingly, MTS and the other simple metabolic diseases (such as hyperglycemia, dyslipidemia, hypertension and T2D) that show abnormalities either in glucose or lipid metabolism are interrelated. It is probably because that the occurrence of MTS and other relevant metabolic diseases involves various mutually dependent pathways and complex interactions between various molecules. Previously, we utilized quantitative endogenous peptidomics analysis to investigate the molecular characteristics of T2D and prediabetes, multiple disease-specific differential peptides were identified and some shared peptide features were also observed 9. However, a general perspective on the overall relationships among MTS and different types of simple metabolic diseases is still lacking. We wonder whether there is an alternative molecular classification of the closely-related metabolic diseases.

The integration of multi-omics data has recently been demonstrated to promote understanding of the development and progression mechanisms of diseases, including cancer 10, obesity 11, T2D 12 and many other diseases 13. Multi-omics models benefit from the simultaneously measurement of multiple relevant bio-molecules in the investigated system. Regarding the metabolic diseases, such important bio-molecules include metabolites, which directly reflect the metabolic state of cells 14. In addition, proteins like p53 15 can regulate the amount of metabolites, and peptides are generated from protein degradation or modification, these two types of molecules can also contribute to metabolic changes. In this context, multi-omics profiling including peptidomics, proteomics and metabolomics enables a meaningful map of molecular changes in the metabolic diseases.

Here, we propose the use of a multi-omics-based framework that integrates metabolomics, proteomics, peptidomics, and clinical information to unveil the latent molecular characteristics and mutual relationships of multiple metabolic diseases, including MTS, obesity, hyperglycemia, dyslipidemia, hypertension and T2D. Our study revealed a number of important findings, including (1) identification of shared and disease-specific molecular patterns, (2) an alternative metabolic disease subtyping pattern which showed significant molecular and clinical differences in glucose and lipid metabolism, (3) different compensatory and molecular regulation mechanisms underlying the redefined metabolic subtypes were observed. This work helps to further our understanding of the intra-disease heterogeneity and inter-disease similarity underlying the existing classification, provides an alternative disease classification which is mainly linked to disparation in lipid and glucose metabolism, thus improving the early diagnosis and treatment of MTS relevant metabolic diseases.

## Results

### Overview of the multi-omics workflow

**Figure 1:**
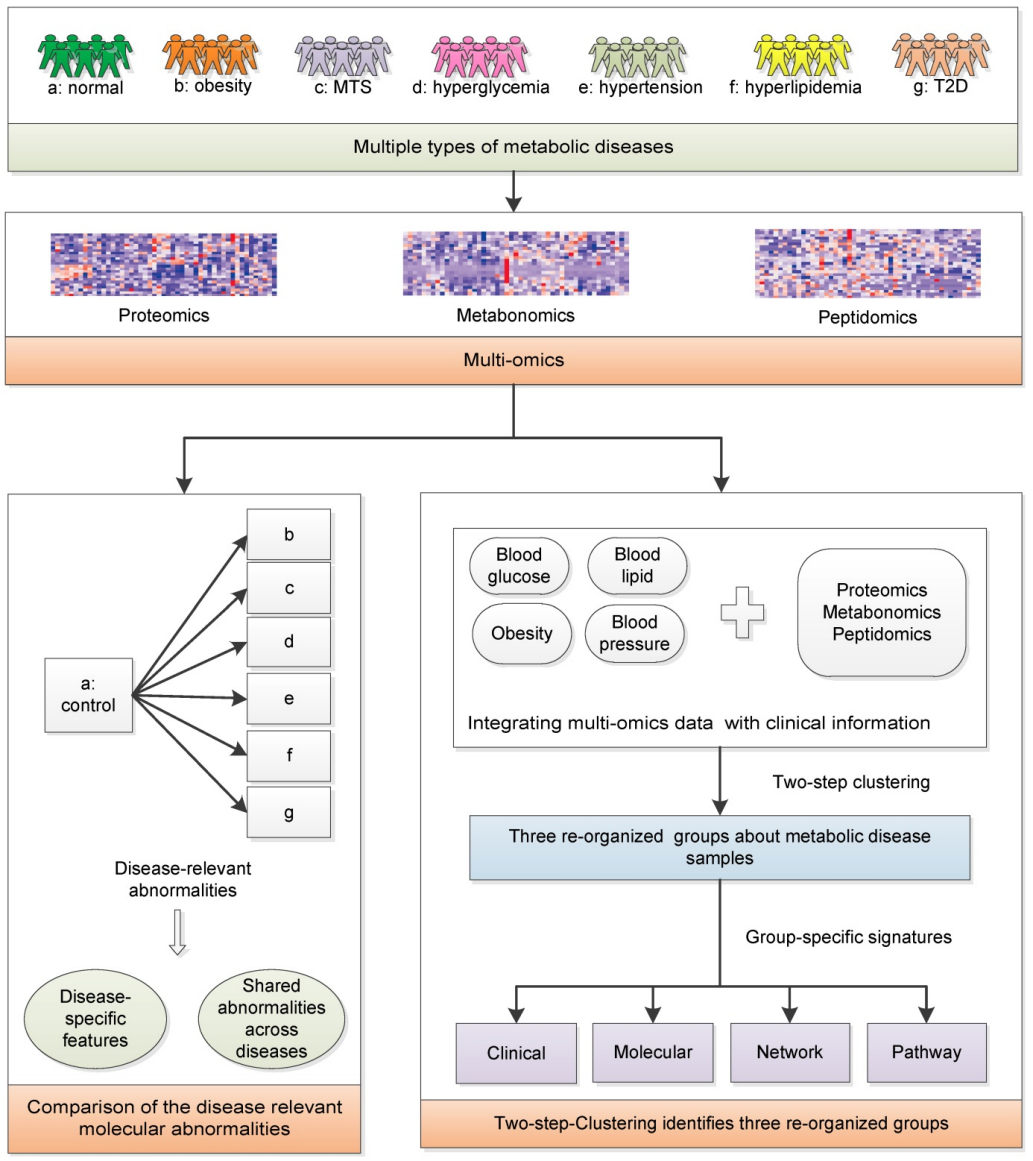
The systematic framework. MTS: metabolic syndrome; T2D: type 2 diabetes.

We aim to achieve two goals: (1) investigate the relationships among MTS and relevant metabolic diseases considering their molecular characteristics; (2) redefine the molecular classification of the investigated metabolic diseases. Serum samples from healthy individuals and patients with metabolic diseases were collected: Group a-healthy individuals; Group b-simple obesity/overweight; Group c-MTS; Group d-simple hyperglycemia, Group e-simple hypertension, Group f-simple hyperlipidemia; Group g-simple T2D (n=7 for each group, Figure 1). Their basic demographic characteristics are summarized in Table 1 and group-specific distributions of the most decisive clinical factors are shown in Figure S1. The clinical diagnostic criteria for the six types of diseases 16 are listed in Table 2. To ensure that the collected samples were only influenced by the expected diseases, the clinical symptoms of each patient were strictly checked, e.g., the hyperglycemia patients only had abnormal blood glucose factors but not other factors (Figure S1). Next, metabolomics, proteomics and peptidomics approaches were employed to measure the molecular profiles of the collected samples.

We compared the multi-omics profiles of each disease group to that of the healthy group and identified the differentially expressed molecules (DEMs). Then, we analyzed the results to further identify the disease-specific and multi-disease-shared molecular abnormalities among the six types of metabolic diseases.

Considering the high heterogeneity of metabolic diseases, it is essential to investigate whether there is an alternative way to classify these metabolic diseases. All clinical samples were clustered based on both multi-omics data and clinical information. Differences in the clinical and molecular patterns among the three groups were identified. Pathway and network analyses were applied to help reveal the distinctive biological processes underlying the identified disease groups.

### Multi-omics profiling reveals both shared and disease-specific molecular characteristics

**Figure 2:**
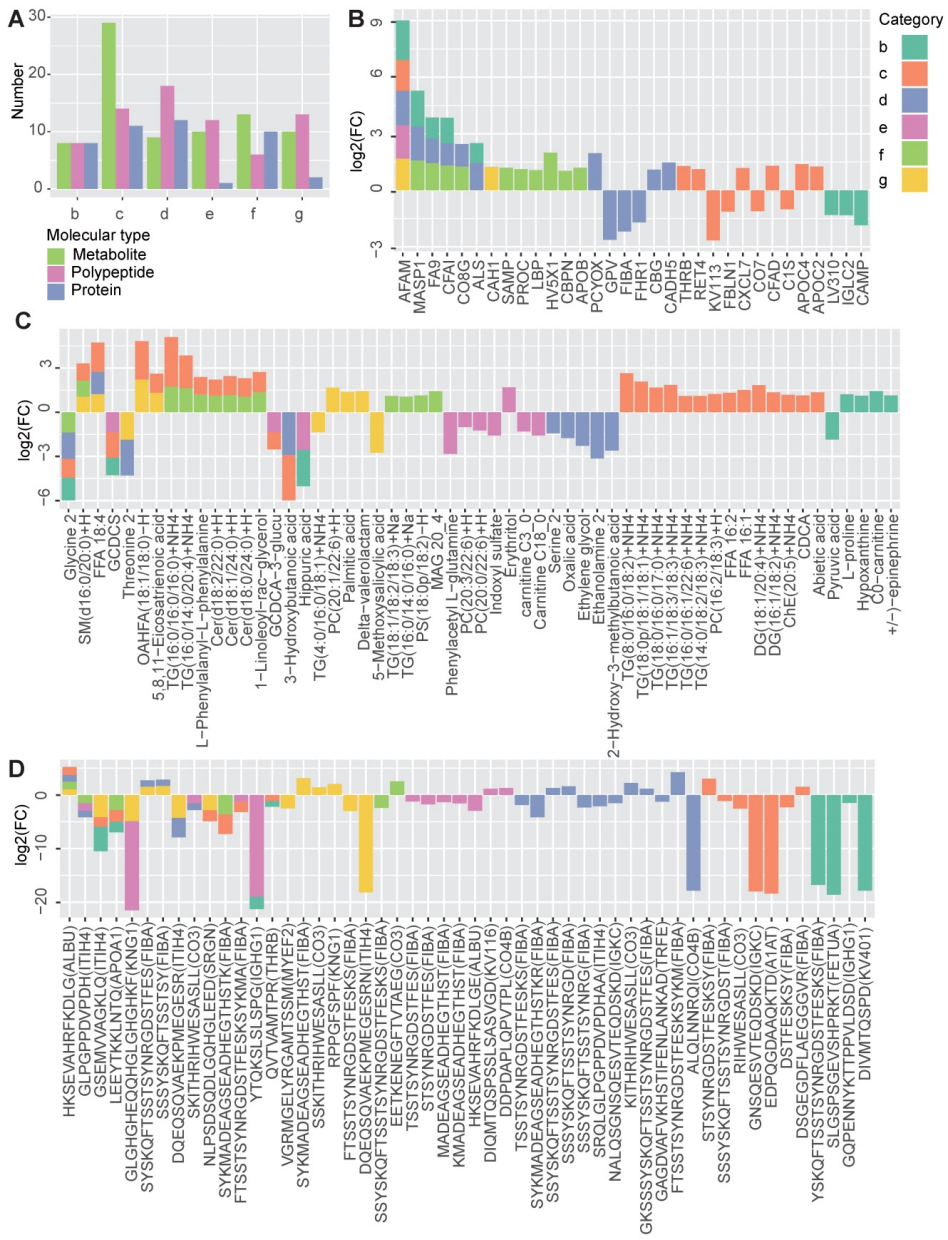
Shared and specific molecular features of the metabolic diseases. **A** The number of differentially expressed metabolites, polypeptides and proteins obtained by comparing each disease group (B to G) to the normal group. **B-D** The profiles of the shared and specific alterations for all disease groups in terms of proteins (B), metabolites (C) and polypeptides (D). The bar colors represent the disease groups. The vertical axis represents the log2-changed fold change between two groups (the mean value of the disease group divided by that of the normal group), while the horizontal axis represents different molecules. The source protein names for the polypeptides are annotated in the brackets after the polypeptide names. n =7 for each group.

According to the multi-omics profiling data, each disease group showed numerous differentially molecules, reflecting the molecular characteristics of the diseases (Figure 2A). Regardless of the molecular type, MTS had the largest number of DEMs (n = 54, group c), due to the fact that MTS is more complicated than its component metabolic diseases, and it is not simply the addition of multiple metabolic diseases. The hyperglycemia (n = 39, group d) and hyperlipidemia (n = 29, group f) groups were next, and the hypertension group (n = 23, group e) had the lowest number of DEMs.

By comparing the DEMs identified in the different groups, both shared and disease-specific DEMs were observed (Figure 2B-2D). At the proteomic level, obesity and hyperglycemia (groups b and d) showed the largest number of shared DEMs (Figure 2B). At the metabolomic level, MTS and hyperlipidemia (groups c and f) shared the largest number of DEMs (Figure 2C). At the peptidomic level, however, hyperglycemia and T2D (groups d and g) shared the largest number of DEMs (Figure 2D).

Some of these shared molecules have been previously reported (Table S1). Afamin (AFAM) is a human plasma vitamin E-binding glycoprotein. Its plasma concentration has been found to be highly associated with MTS 17 and insulin resistance 18. In this study, we further confirmed that the AFAM level was higher in both MTS and most of the other simple metabolic diseases, including obesity, hyperglycemia, hypertension and T2D. Mannan-binding lectin serine protease 1 (MASP1), which plays a role in the lectin pathway of the complement system, has been identified as a biomarker of prediabetes and is relevant to obesity, dyslipidemia and hypertension in cardio- and cerebrovascular patients 19. In this study, the significantly increased expression of MASP1 was observed in patients with simple obesity, hyperglycemia and hyperlipidemia (group b, d and f). Some studies have found a negative association between the level of serum glycine and several metabolic diseases, such as T2D 20, obesity 21, MTS 22 and hyperlipidemia 23. Our results showed that glycine levels were decreased in MTS, obesity, hyperglycemia and hyperlipidemia patients. Ceramide (Cer) is known to participate in the pathogenesis of insulin resistance and other obesity-associated metabolic diseases 24; however, Cers [Cer(d18:0/24:0)+H, Cer(d18:1/24:0)+H, Cer(d18:2/22:0)+H] was increased only in MTS and hyperlipidemia patients when compared to that in patients with other simple metabolic disorders (Figure 2C).

Disease-specific DEMs were also observed. MTS had the largest number of disease-specific DEMs. Different types of lipids, including diacylglycerol (DG), TG, free fatty acids (FFA), and phosphatidylcholine (PC) showed specifically increased expression in MTS patients (Figure 2C). Correspondingly, lipid regulating proteins such as apolipoprotein C-II (APOC2) and thrombin (THRB) 25, 26 were also specifically altered in MTS patients (Figure 2B). Except of lipid metabolism relevant molecules, proteins involved in immune system such as complement factor D (CFAD), complement component C7 (CO7) and immunoglobulin kappa variable 1-13 (KV113) 27 (Figure 2B), and polypeptides from proteins relevant to immunity, including immunoglobulin kappa constant (IGKC), fibrinogen alpha chain (FIBA) and complement factor C3 (CO3), were also altered in MTS (Figure 2D). These molecules showed significant alterations only for MTS but not for other metabolic diseases, implying the importance of lipid metabolism and immune response in MTS.

More disease-specific DEMs were identified than shared DEMs, implying the presence of significant differences among these diseases (Figure 2B-2D). Some of the DEMs associated with specific diseases and the relationships between them have been revealed by previous studies (Table S2). For hyperglycemia, specific DEMs including cadherin-5 (CADH5) 28, corticosteroid-binding globulin (CBG) 29, and a polypeptide from serotransferrin (TRFE) 30 have been reported to be associated with either elevated blood glucose, T2D or insulin resistance. However, in our study, we found that their expression levels showed significant changes in the prediabetes group (group D) but not in the T2D or MTS group. In contrast, carbonic anhydrase 1 (CAH1) and polypeptides from myelin expression factor 2 (MYEF2) 31 and kininogen-1 (KNG1) 32 showed specific abnormalities only in T2D but not in prediabetes.

### The investigated patients were stratified into three main groups by integrating multi-omics data and clinical information

**Figure 3:**
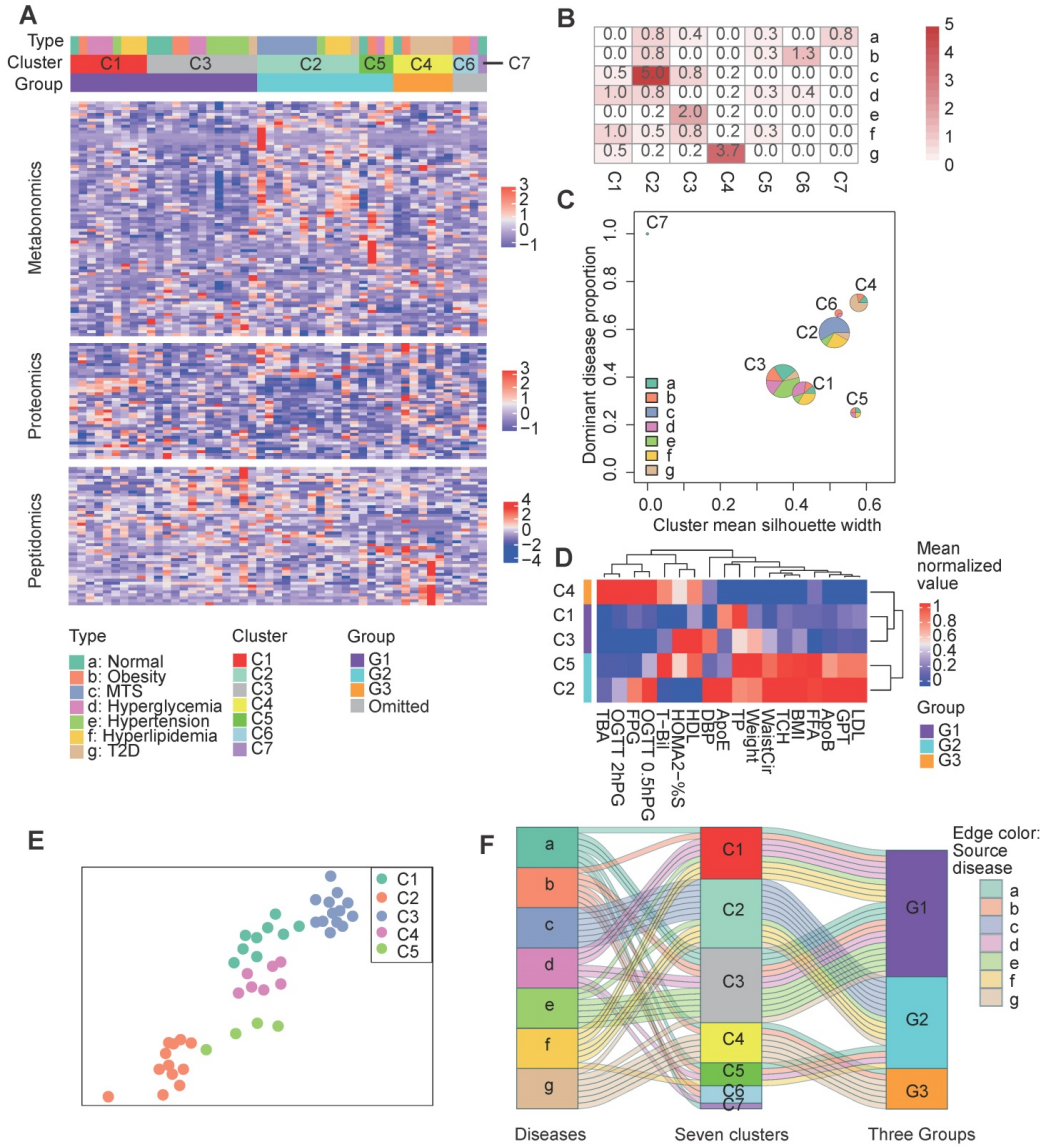
Clustering of patients based on both clinical factors and multi-omics data. **A** Clustering results. The central heatmap displays the normalized expression of clinical factor-relevant molecules (see Methods) according to metabolomics, proteomics and peptidomics, in which each column corresponds to a patient and each row corresponds to a molecule. Above the heatmap, the three rows indicate the original patient groups (A to G), the first-step clustering results (seven clusters: C1 to C7) and the three combined groups (G1 to G3). See the Methods for the detailed clustering methods. **B** Significance of the overlaps between the different clusters (represented by the columns) and the patient groups (represented by the rows) according to Fisher's exact test. **C** Visualization of the cluster composition and homogeneity. Each pie represents the disease-type composition within an individual cluster, and the size is proportional to the number of samples. The x and y coordinates represent the cluster silhouette width and the proportion of the most dominant disease type within a cluster, respectively. **D** The second step clustering results. The heatmap displays the mean cluster levels in a collection of clinical factors. **E** The force-directed map layout was computed from a combined similarity matrix calculated as the dot product of the consensus clustering matrix and a differential clinical factor-based Spearman correlation matrix for the samples, and similar samples are positioned close to each other. **F** The Sankey diagram describes the relationships between the original disease types, the initial seven clusters and the three combined groups. C1, n= 9; C2, n=12; C3, n=13; C4, n=7; C5, n=4; C6, n=3; C7, n=1. 0.5hPG: 0.5-hour postprandial plasma glucose; 2hPG: 2-hour postprandial blood glucose; ApoB: apolipoprotein b; ApoE: apolipoprotein e; BMI: body mass index; DBP: diastolic blood pressure; FFA: free fatty acids; FPG: fasting plasma glucose; GPT: glutamic pyruvic transaminase; HDL: high density lipoprotein; HOMA2: homoeostasis model assessment 2; LDL: low density lipoprotein; MTS: metabolic syndrome; OGTT: oral glucose tolerance test; %S: insulin sensitivity index; T2D: type 2 diabetes; TBA: total bile acid; T-Bil: total bilirubin; TP: total protein; WaistCir: waist circumference.

The above analysis indicates the molecular commonness and specialty of different metabolic diseases. We wonder whether there is an alternative disease classification way that the redefined groups show more obvious molecular and clinical separations. Initially, we attempted to cluster the samples simply based on the multi-omics data (Figure S2A). However, most of the diseases were re-organized into disparate clusters, few of the identified clusters were enriched by one dominant disease type (Figure S2B, only the cluster C1 was enriched by the hyperglycemia disease at the significant level p<0.01). Therefore, we wonder whether integrating the multi-omics data with clinical information can help improve the results. We re-clustered the collected samples with an unsupervised two-step clustering method which combined multi-omics data and clinical information (see Methods). For the first step clustering, instead of clustering based on all items within the multi-omics data, we filtered the molecules based on their correlations with the key clinical factors and only the key clinical factor relevant molecules (see Methods) were retained for the clustering analysis. As results showed, the samples were clustered into seven new groups (Figure 3A). The clustering results showed remarkable differences upon comparison to the original disease groups (Figure 3A). Notably, more clusters were predominated by certain disease types, e.g., cluster 2 (C2) was enriched in MTS patients (p=1e-5), cluster 3 (C3) was enriched in hypertension patients (P=1e-2), and cluster 4 (C4) was enriched in T2D patients (P=1e-3.7) (Figure 3B). However, most clusters comprised a mixture of disease types. This challenges the present metabolic disease classification, treatment or diagnosis based only on the routine disease classification may be insufficient. For example, two obesity patients respectively having similar molecular profiles with the MTS and T2D patients should be treated differently.

To further characterize the relative composition and homogeneity of these clusters, we computed the proportion of the dominant disease type and the mean silhouette width 10 for each cluster (Figure 3B). The single disease type dominant clusters, such as C2 and C4, had relatively increased silhouette widths, suggesting higher within-cluster homogeneity for these clusters (Figure 3C). Although each sample in cluster 5 (C5) comprised a different disease type, C5 still had a high silhouette width, implying the presence of inter-disease similarity in certain patients and the robustness of the clustering of C5.

During the second step clustering (see Methods), we further clustered the first five main clusters (the number of samples in C6 and C7 was so small that they were not considered for the following analysis) based on their mean clinical factor values (Figure 3D), we observed that C1 & C3 or C2 & C5 had more mutual similarities, while C4 was relatively different. A force-directed graph layout-based mapping (performed by the “layout_with_drl” function in the R package igraph 33) also indicated that the C1 and C3 and the C2 and C5 samples were more closely related to each other (Figure 3E). Consequently, we merged C1 and C3 as well as C2 and C5 into two larger groups, termed G1 and G2, respectively, and defined C4 as the third group, G3.

To better understand the relationships between the original disease groups and redefined groups, we visualized the relationships with a Sankey-plot (Figure 3F). A large proportion of the hypertension (6 out of 7), hyperglycemia (5 out of 7) and normal (4 out of 7) samples were categorized into the G1 group. G2 was mainly composed of MTS (all 7) and hyperlipidemia samples (4 out of 7). G3 was enriched in T2D patients. Differences in the predominant disease types in the groups indicate the presence of heterogeneity across the identified groups; in addition, the co-occurrence of multiple disease types in the same group implies similarity between disease types. In short, the across-disease clustering result reflects the intra-disease heterogeneity as well as the inter-disease similarity of these metabolic diseases.

### The redefined groups exhibited remarkable differences in glucose and lipid metabolism

**Figure 4:**
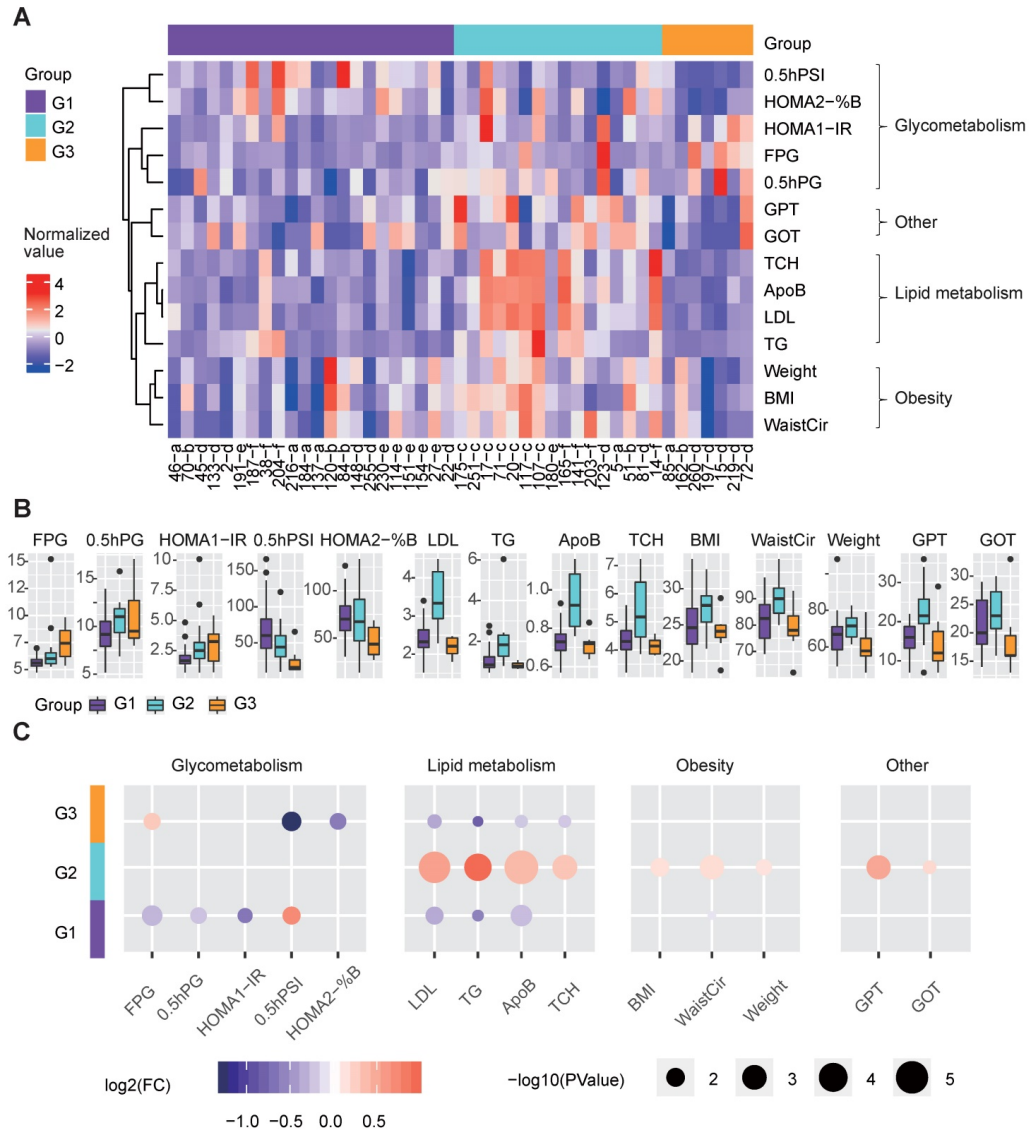
Clinical features of the three metabolic disease groups. **A** The heatmap shows the normalized clinical factor levels for the samples in the three main groups. Only clinical factors showing significant differences between one specific group and the others are shown. **B** The box plots represent the clinical factor levels in the three groups. The centers of the boxes represent the median values. The bottom and top boundaries of the boxes represent the 25th and 75th percentiles. The whiskers indicate 1.5 times of the interquartile range. The dots represent points falling outside this range. **C** The circle colors represent the log2-transformed fold change of certain clinical factors between one group and the others. The circle size is proportional to the -log10(P) value; the P values were calculated by the Wilcox test (two-sided, unpaired) to compare the differences between one specific group and the others. G1 was compared to the combination of G2 and G3, G2 was compared to the combination of G1 and G3, and G3 was compared to the combination of G1 and G2; the same as below). 0.5hPG: 0.5-hour postprandial plasma glucose; 0.5hPSI: 0.5-hour postprandial serum insulin; ApoB: apolipoprotein b; %B: pancreatic islet b cell function index; BMI: body mass index; FPG: fasting plasma glucose; GOT: glutamic oxalacetic transaminase; GPT: glutamic pyruvic transaminase; HOMA2: homoeostasis model assessment 2; IR: insulin resistance index; LDL: low density lipoprotein; TCH: total cholesterol; TG: triglyceride; WaistCir: waist circumference.

We further examined whether clinical differences were still detectable based on the three redefined groups. These three groups showed significant differences in terms of clinical factors related to glucose and lipid metabolism (Figure 4A-4B).

G3 showed more seriously dysregulation in terms of glucose metabolism, as its mean FPG level was the highest, and its mean 0.5-hour postprandial serum insulin (0.5hPSI) and homoeostasis model assessment (HOMA) 2 estimate of β-cell function (HOMA2-%B) were much lower than those in the other groups (Figure 4B-4C). G1 had significantly decreased levels of FPG, 0.5-hour postprandial blood glucose (0.5h PBG), and HOMA 1 estimate of insulin resistance (HOMA1-IR), and an increased level of 0.5hPSI compared to G2 and G3 (Fig. 4b-4c).

With respect to lipid metabolism, G2 had remarkably increased levels of several lipid metabolism-relevant factors, including LDL, TG, apolipoprotein B (ApoB) and total cholesterol (TCH) (Figure 4B-4C), implying that G2 was mainly dysregulated in terms of lipid metabolism.

Additionally, G2 showed significantly higher glutamic pyruvic transaminase (GPT) and glutamic oxalacetic transaminase (GOT) levels than the other groups, indicating that the liver function of patients in G2 were more likely to be impaired than the other groups (Figure 4B-4C). Meanwhile, G2 also showed significantly increased levels of obesity-relevant clinical factors, especially the WaistCir value (Figure 4B-4C).

Overall, the three redefined metabolic disease subtypes, although different from original disease classification, showed remarkable and specific differences in terms of clinical characteristics. G2 was characterized by abnormal lipid metabolism and overweight, and the liver function in G2 patients was worse than that in the other subtypes. G3 mainly showed the serious dysregulation of glucose metabolism. G1 showed relatively favorable clinical characteristics compared to the other subtypes in terms of both glucose and lipid metabolism.

### Multi-omics based molecular signatures of the three groups

**Figure 5:**
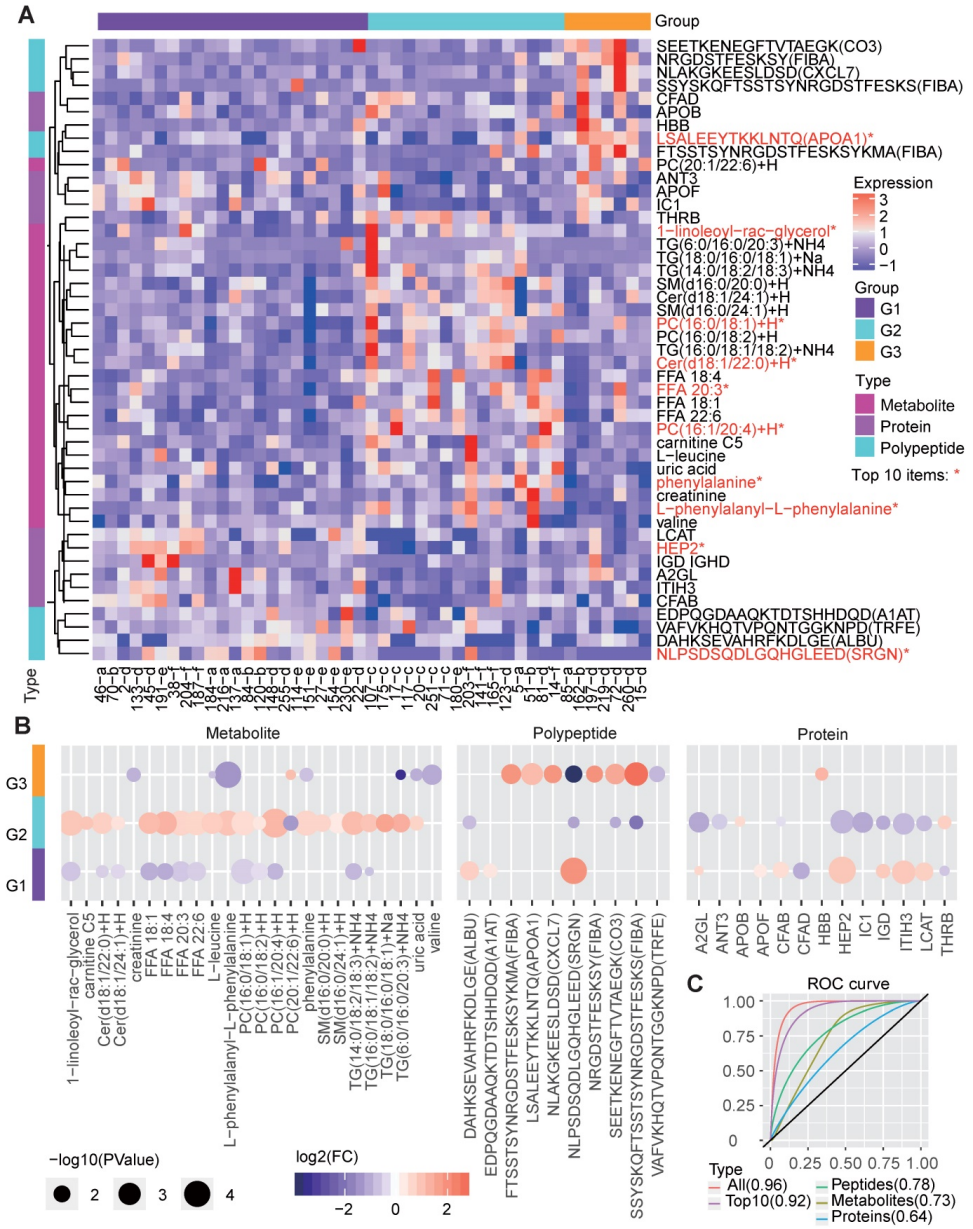
Molecular features of the three metabolic disease groups. **A** The heatmap shows the multi-omics molecular profiles for the samples in the three main groups. The important scores of the molecules in predicting the group labels were computed based on the random forest method, and the differences among groups were examined by the Wilcox test (two-sided, unpaired). Only molecules ranked within the top 50 based on the important scores and showing significant differences (P<0.05) between one specific group and the others are shown, the top-10 ones were marked by red *. The corresponding molecular type (metabolite, protein or polypeptide) is annotated on the left side of the heatmap. **B** The circle colors represent the log2-transformed fold change of a certain molecule between one group and the others. The circle size is proportional to the -log10(P) value, for which the P values were calculated by the Wilcox test (two-sided, unpaired) to compare the differences between one specific group and the others. C ROC curves about the performance of molecular profiles to predict the re-defined groups. We respectively utilized the identified group-differential metabolites, proteins, polypeptides, all three types of molecules in B and the top-10 important ones among them to train the classifiers, and four corresponding ROC curves were drawn. The AUCs are given in the brackets in the curve legend. AUC: area under curve; ROC: receiver operating characteristic.

The corresponding molecular characteristics in the redefined groups were also described (Figure 5A). We applied random forest analysis to estimate the importance of various molecules in identifying the three groups and examined the expression differences between groups based on the multi-omics data. Among the top 50 most important molecules, 47 ones, including 24 metabolites, 10 polypeptides, and 13 proteins, also showed significant differences between the groups (Figure 5A-5B).

G1 showed significantly decreased levels of several lipid metabolites, which is consistent with the clinical features resulting from low lipid metabolism levels. Correspondingly, G1 also showed increased levels of proteins such as lecithin cholesterol acyltransferase (LCAT), which contributes to HDL biogenesis 34, and complement factor B (CFAB) which plays a role in the complement system 35, and two polypeptides from serglycin (SRGN) and serum albumin (ALBU) (Figure 5B). These lipid-regulating and immune relevant proteins or peptides may help reduce the blood lipid level.

G2, in line with the revealed dysregulation in lipid metabolism, showed significantly increased levels of multiple lipid metabolites, such as the different forms of TG, PC, FFA, Cer and 1-linoleoyl-rac-glycerol. In contrast, several proteins, such as Heparin cofactor 2 (HEP2), CFAB and LCAT, were significantly decreased in G2 (Figure 5B).

With respect to G3, molecular differences were mainly found in the polypeptides, especially those involved in the immune response, such as apolipoprotein A-I (APOA1) 36, FIBA 35 and CXCL7 37. In addition, G3 had decreased levels of certain metabolites (Figure 5B), such as phenylalanine, creatinine, valine and TG. Previous studies have shown that phenylalanine is associated with T2D pathophysiology 38 and that a low serum creatine level is a risk factor for T2D 39. Therefore, we deduce that G3 patients are either with T2D or at high risk of T2D.

The remarkable molecular differences found between different groups reveal the potential molecular basis for the three clinically distinct groups. We further examined the performance of these molecules in predicting the redefined groups, and the corresponding receiver operating characteristic (ROC) curves were drawn (Figure 5C). As the results showed, the integrated three types of molecular features (area under ROC curve [AUC] = 0.96) were much better than each single type of molecules (AUC=0.64, 0.73 and 0.78 for classifiers only based on proteins, metabolites and polypeptides) in predicting the right groups, and even the top-10 important multi-omics elements (including 1-linoleoyl-rac-glycerol, PC, HEP2 and peptide from SRGN, etc., Figure 5A) can generate a classifier with a relatively good performance (AUC=0.92, Figure 5C).

### Heterogeneous network dynamics in the three groups

**Figure 6:**
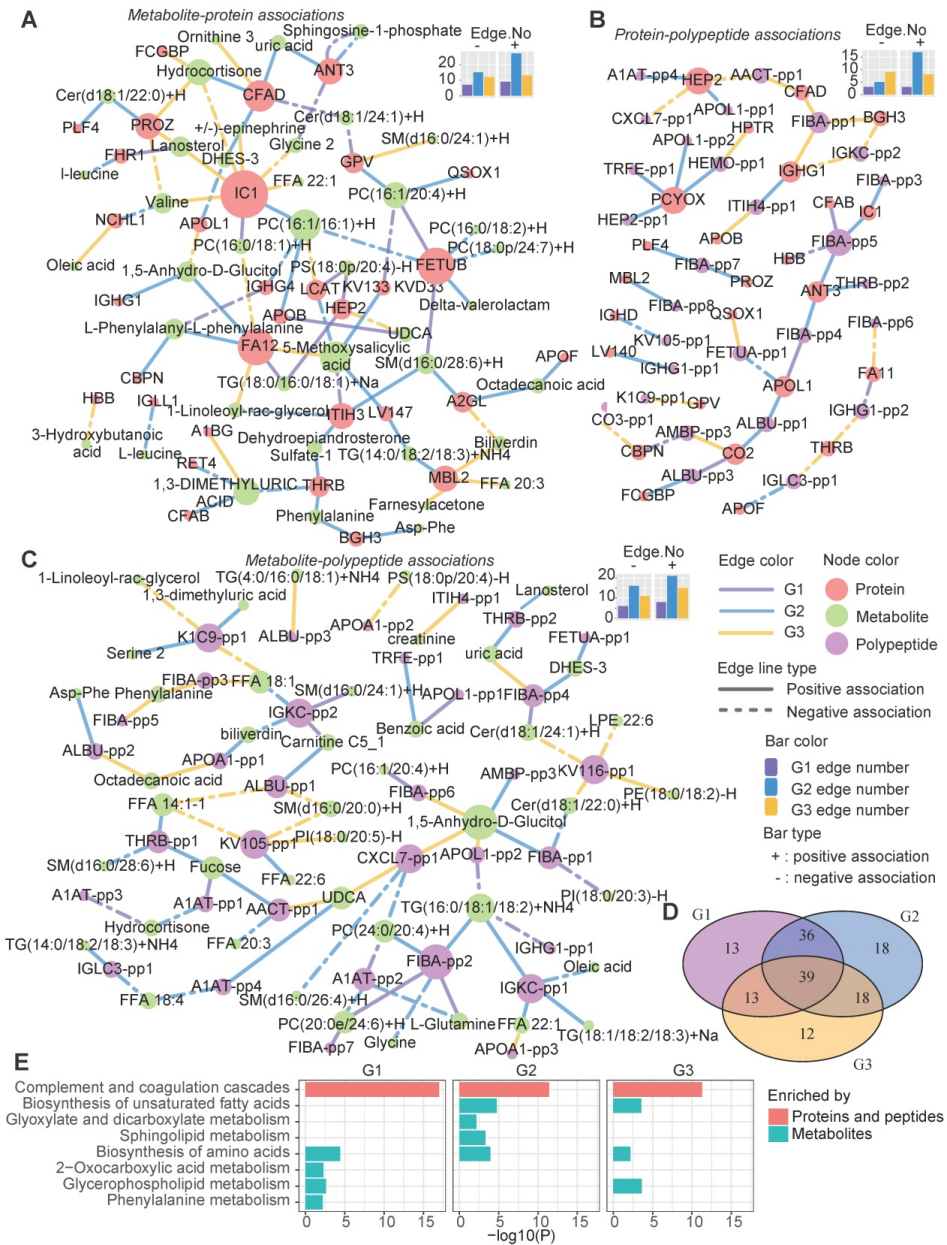
Heterogeneous molecular network dynamics in the three metabolic disease groups.** A-C** Significant associations between metabolites and proteins (A), proteins and peptides (B), and metabolites and peptides (C) in the G1, G2 and G3 samples (absolute value of the correlation coefficient > 0.6, P < 0.01). The associations were estimated by biweight midcorrelations, and the corresponding Student p-values were calculated. The node colors represent the molecular types. The solid and dashed lines represent positive and negative correlations, respectively, between the connected nodes. The edge colors indicate in which groups the associations were found. The top-right bar plots summarize the positive (+) and negative (-) edge numbers (Edge.No) in different groups. For clarity, the peptides are represented by abbreviated names; see Table S3 for the detailed peptide information. **D** The overlap among the network nodes for different groups. **E** Pathway enrichment analysis of the network nodes for different groups. The bar lengths are proportional to -log10(P), and the bar colors indicate whether the pathways were enriched in proteins (both proteins and source proteins of the peptides) or metabolites.

We also assessed the variations of molecular interactions that can reflect the molecular compensatory mechanisms in different groups. Different from previous studies which mainly focus on the gene network, here, three types of heterogeneous molecular networks, the metabolite-protein (Figure 6A), polypeptide-protein (Figure 6B), and metabolite-polypeptide (Figure 6C) association networks, were constructed. LCAT can catalyze the conversion of PC to 1-acyl-sn-glycero-3-phosphocholine. Consequently, LCAT and PC may be negatively correlated. Here, we observed that LCAT showed a significant negative association with PC in G3 (Figure 6A); however, this association disappeared in G1 and G2. Instead, LCAT showed a positive and a negative association with phosphatidylserine (PS) and 5-methoxysalicylic acid in G1 and G2, respectively (Figure 6A), suggesting the influence of disease subtype-specific regulation mechanisms. Different groups exhibited distinct edges, although the nodes were highly overlapped (Figure 6D), suggesting that the differences in the networks among the groups were mainly dependent on the dynamic associations between molecules rather than the molecules themselves. G2 was observed to have the largest number of positive edges in all three types of networks, while G1 had the minimum number of edges (both positive and negative). G3 had the maximum number of negative edges in the protein-polypeptide association network.

A pathway enrichment analysis showed that the cross-talk between the immune and metabolism systems contributed to the metabolic disease subtypes (Figure 6E). The proteins (both the proteins themselves and the source proteins of the peptides in the networks) in these networks were mainly involved in the complement and coagulation cascade pathways (Figure 6E).

Meanwhile, both shared and group-specific metabolic pathways were observed among the groups (Figure 6E). For instance, the metabolic nodes in the networks were significantly enriched in the “biosynthesis of amino acids” pathway in all three groups, and both G1 and G2 were enriched in “glycerophospholipid metabolism”. G2 showed specific enrichment of “sphingolipid metabolism” and “glyoxylate and dicarboxylate metabolism”, which is associated with the tricarboxylic acid cycle, and both G2 and G3 were enriched in the “biosynthesis of unsaturated fatty acids” pathway (Figure 6E).

Overall, these three groups also had disparate molecular network characteristics, implying that different compensatory and molecular regulation mechanisms underlie the identified metabolic subtypes, especially in the immune response and lipid metabolism pathways.

### Pathway characteristics of the three groups

**Figure 7:**
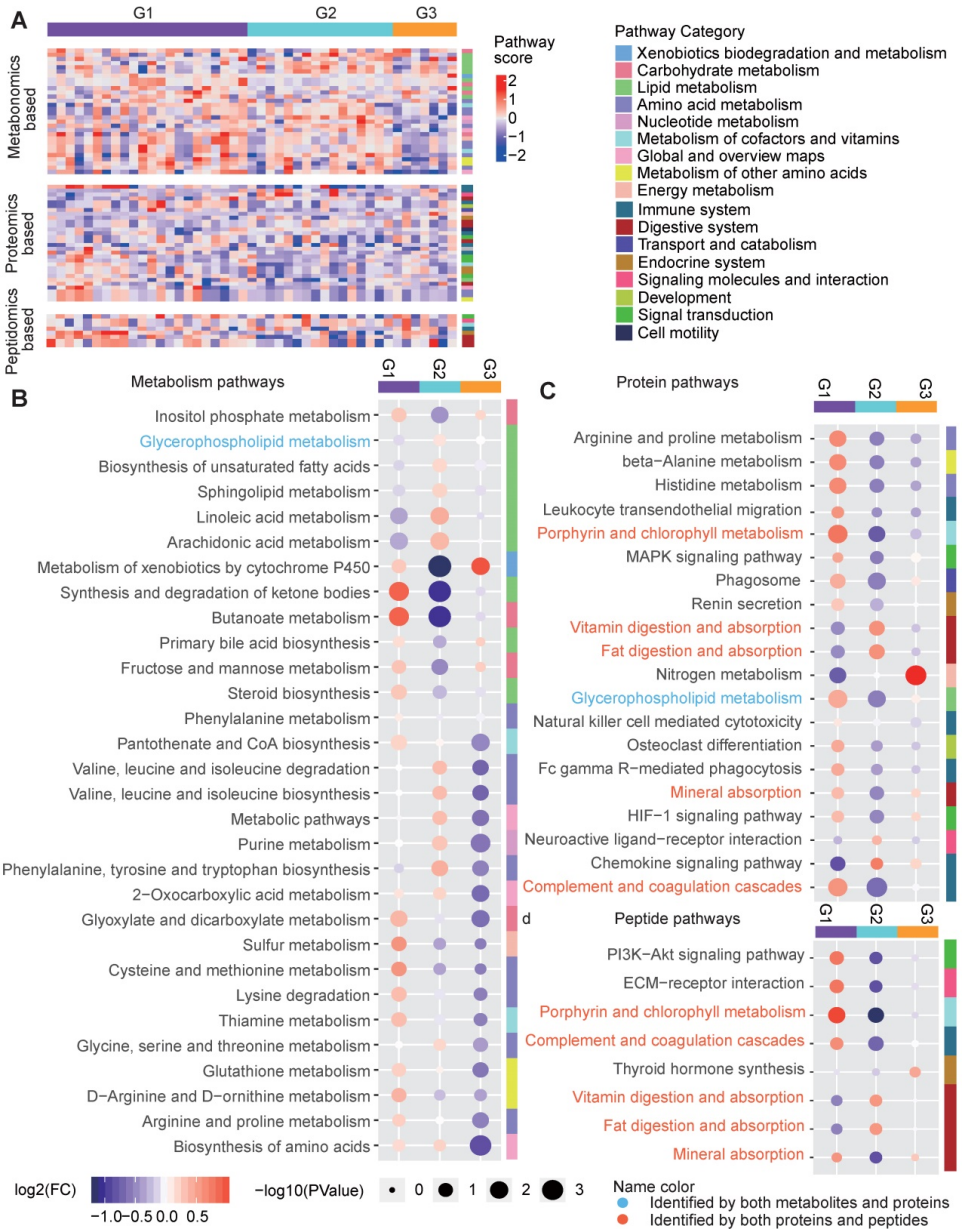
Pathway characteristics of the three metabolic disease groups. **A** Pathway profile of samples in the three main groups. For every pathway, the GSEA method was applied to examine whether the members of the pathway were enriched at the top or bottom of the ranked molecular list for an individual sample with respect to each type of omics data (see Methods). The pathway category is annotated at the right side of the heatmap. **B-D** Top-ranked differential pathways in the three groups identified based on the metabolomics (B), proteomics (C) and peptidomics (D) profiles, respectively. Random forest-based important scores for the pathways were also calculated to estimate the importance of a pathway in predicting the group labels. Then, the top-half ranked pathways (the number of top-half metabolomics-based pathways was larger than 30, so we only displayed the top 30 for clarity) were further examined. The GSEA-based pathway scores in the samples in each pathway were normalized by subtracting the minimum level. The circle colors represent the log2-transformed fold change of the normalized GSEA scores for a certain pathway between one group and the others. The circle size is proportional to the -log10(P), for which the P values were calculated by the Wilcox test (two-sided, unpaired). GSEA: gene set enrichment analysis.

We also characterize the pathway features of different groups. For each type of omics-data, we calculated the Gene Set Enrichment Analysis (GSEA)-based pathway activity scores (see Methods) for each sample for all calculable Kyoto Encyclopedia of Genes and Genomes (KEGG) pathways and compared the scores among the three groups (Figure 7A). Remarkable differences in the pathway scores were found (Figure 7B-7D). G1 was characterized by much lower metabolic levels in lipid metabolism pathways, such as the “glycerophospholipid metabolism”, “biosynthesis of unsaturated fatty acids”, “sphingolipid metabolism”, “linoleic acid metabolism” and “arachidonic acid metabolism” pathways, and this may be related to increased enzyme levels in lipid metabolism pathways, as indicated by high scores in proteomics-based pathways such as “glycerophospholipid metabolism” (Figure 7B-7D). However, in addition to increases in the activity of lipid metabolism pathways, G1 was mainly characterized by the up-regulation of different types of pathways, including “synthesis and degradation of ketone bodies”, “butanoate metabolism”, “glyoxylate and dicarboxylate metabolism”, “cysteine and methionine metabolism”, “complement and coagulation cascades”, and “porphyrin and chlorophyll metabolism”, regardless of the omics data type (Figure 7B-7D), suggesting the hyperactivity of metabolism and immune responses in the G1 samples. G2 showed a pathway profile that was almost reversed comparing to G1, in which most of the lipid metabolism pathways and amino acid metabolism pathways for phenylalanine, tyrosine and tryptophan, and valine, leucine and isoleucine were assigned increased scores in G2, and the proteomics-based pathway scores for most metabolic and immune system-relevant pathways were significantly decreased compared to those in the other groups. Meanwhile, the digestive pathways for vitamins and fat were up-regulated in G2 based on the proteomics data (Figure 7B-7D), suggesting that the metabolites generated from the hyperactive digestive system might be excessively accumulated in the G2 samples. Compared to G1 and G2, G3 did not show significant differences in the lipid metabolism pathways; however, its metabolomics-based pathway scores for most amino acid, nucleotide, cofactor and vitamin metabolic pathways were decreased (Figure 7B), and its proteomics-based pathway scores for “nitrogen metabolism” were significantly increased (Figure 7C), implying that the presence of dysregulated glucose metabolism in G3 might be related to the amino acid, nucleotide or nitrogen metabolism pathways. In addition, we observed that the peptidomics-based results were generally consistent with those obtained from the proteomics data (Figure 7C-7D).

## Discussion

A comprehensive description of the molecular and clinical characteristics of different metabolic diseases can promote understanding of the relationships among various metabolic diseases. In this study, we integrated three omics data analyses and clinical information from patients to investigate the molecular characteristics of several commonly occurring and closely related metabolic diseases, including obesity, hyperglycemia, hyperlipidemia, hypertension, MTS and T2D.

Both the shared and specific molecular profiles for the six types of metabolic diseases including MTS and relevant diseases were identified. The shared molecular features imply the progressive possibility between different metabolic diseases, and also suggest the present disease classification does not have clear molecular separation. The routine diagnosis and treatment of these metabolic diseases might overlook the connection and heterogeneity of these closely-related diseases.

To further investigate whether there is an alternative way to stratify these metabolic disease patients, we redefined three disease groups through a two-step clustering analysis which integrates both multi-omics data and clinical information. Although the clustering results were distinct from the original disease definitions, the newly clustered groups exhibited distinctive patterns from both clinical and molecular perspectives. The first group (G1) was composed of the most heterogeneous metabolic disease samples; however, no MTS patients and only one T2D patient were included in G1, and all of the patients in G1 showed significantly more favorable levels in terms of clinical factors relevant to both glucose and lipid metabolism, indicating a lower likelihood for G1 patients to progress to metabolic syndrome or T2D. G2 was mainly enriched in MTS and hyperlipidemia patients, and they showed significantly higher levels of lipid metabolites and corresponding clinical factors. G3, a group enriched in T2D patients, was mainly characterized by the dysregulation of glucose metabolism. Although G2 and G3 were predominated by MTS and T2D, respectively, there were many simple metabolic disease patients spreading among the three groups, and one hyperglycemia or hyperlipidemia patient could be classified as G1-, G2- or G3-like according to our subtyping strategy. This indicates the possibility that simple metabolic diseases could progress into more complex diseases, i.e., MTS or T2D, thus providing clinical or molecular clues useful for early disease prevention. For instance, G2-like hyperlipidemia patients might be more likely to progress to MTS than G1-like hyperlipidemia patients.

Meanwhile, the underlying molecular signatures, molecular regulation networks and pathways in these three groups were completely distinct, suggesting the different compensatory and molecular regulation mechanisms underlying the refined metabolic subtypes, especially with respect to lipid metabolism, amino acid metabolism and the immune system. Notably, these group specific features were from different molecular sources, single type of omics data cannot grasp the distinctive patterns.

We cannot claim that the new groups identified here are better than the previous disease classification. But these re-organized groups could still provide an alternative way to classify metabolic diseases and to understand the relationships, especially the potential progression among different metabolic diseases. In the future, we will undertake more comprehensive investigations and utilize experimental assays to further explore the specific mechanisms. The group-specific clinical and molecular profiles can provide guidance for the investigation of potential molecular mechanisms and even preventive or diagnostic biomarkers and therapeutic targets, thus improving the treatment and prevention of these highly correlated metabolic diseases.

Our data provided insights into classifying metabolic diseases. The limitations of our study include the small sample size and the lack of genomics analysis, as our initial genomics data did not yield meaningful results. Future larger studies will be conducted to continually improve the results.

## Methods

### Clinical sample collection and ethics committee approval

Serum from 49 individuals and the corresponding clinical information were collected by the Shanghai Jiao Tong University Affiliated Sixth People's Hospital with the approval of the ethics committee. The serum samples were immediately placed on dry ice and mailed to the Dalian Institute of Chemical and Physics, after which they were placed in a -80˚C refrigerator for storage. Group A was comprised of healthy persons, while groups B, C, D, E, F and G were comprised of patients with obesity, MTS, hyperglycemia, hypertension, hyperlipidemia and T2D, respectively each group comprised seven randomly sampled people. During collection, except for the MTS patients (group C), we ensured that the serum samples in each group were obtained from patients with only one of the specified diseases.

### HOMA calculator

HOMA1 was calculated according to the original HOMA model 40. HOMA2 was calculated by HOMA2 Calculator 40 software, for which the fasting plasma glucose and insulin concentrations were utilized for the calculations.

### Metabolome profiling

Metabolomics and lipidomics profiling was performed with a Waters UPLC system coupled with a Q Exactive HF mass spectrometer (Thermo Fisher Scientific, Rockford, IL, U.S.A.) 41, 42. The separation was performed with a 2.1×100 mm ACQUITY^TM^ 1.7 µm C8 column in ESI positive ion mode, and the mobile phase consisted of water with 0.1% formic acid (A) and acetonitrile (B). For the ESI negative ion mode, the separation was performed with a 2.1×100 mm ACQUITY^TM^ 1.8 µm T3 column, and the mobile phase consisted of 6.5 mM ammonium bicarbonate water solution (C) and 6.5 mM ammonium bicarbonate in 95% methanol and water (D). The separation of the lipid metabolites was performed with a Waters UPLC C8 ACQUITY column (2.1 mm × 100 mm × 1.7 µm) (Milford, MA, USA). GC-MS analysis was also performed for the metabolic profiling. A QP 2010 GC-MS system (Shimadzu, Japan) with a DB-5 MS fused silica capillary column (30 m × 0.25 mm × 0.25 μm, Agilent Technologies, USA) was used. A pseudotargeted GC-MS metabolomics method was used as previously reported 43. Quality control (QC) samples were prepared by mixing equal aliquots of serum from each real sample, and a QC samples was run after 8 real serum samples. The reproducibility of the metabolite ions was evaluated with relative standard deviation (RSD%) of the QC samples. In this study, 78.3% of ions had RSD% less than 20%, and 91.1% of ions had RSD% less than 30%. See Supplementary methods for more details.

### Proteome profiling

Each sample was analyzed in technical triplicate with a nano-RPLC-MS/MS on a Q-Exactive MS (Thermo Fisher, CA) coupled with an Easy-nano LC system (Thermo Fisher, CA). The raw data were uploaded into Maxquant (v.1.6.1.0) and searched against the UniProtKB human complete proteome sequence database (release 2017_06, 24,148 entries), and the average profiling result of three technical repeats for each sample was adopted as the final result. The search included cysteine carbamidomethylation as the fixed modification and methionine oxidation and acetylation of protein N-terminal as variable modifications. The searching tolerance for precursor ions was 10 ppm, and that for fragment ions was 20 ppm. Matching between runs with retention time window of 0.7 min and the label free quantitation algorithm were performed. See Supplementary methods for more details.

### Peptidome profiling

The peptide analysis was performed with nano-RPLC-ESI-MS/MS on an LTQ-Orbitrap Elite mass spectrometer coupled with a Dionex UltiMate 3000 RSLC-nano System (Thermo, San Jose, CA). QC samples were prepared by mixing equal aliquots peptides obtained from each real sample, and the remaining individual samples and QC samples were labeled and analyzed according to a stable isotope dimethyl labeling method 44 where QC and real samples were respectively labeled with dimethyl light label and heavy label, equal volume QC sample was added into each of the real sample, and was analyzed by liquid chromatography tandem mass spectrometry. Then, a ratio value of light labeling intensity/heavy labeling intensity was used to quantify each peptide. The acquired raw MS/MS spectra from each sample were searched against the International Protein Index (IPI) human database with the UniProt website using Mascot Version 2.4.1 (Matrix Science). MaxQuant software (version 1.6) was used to perform the quantitative analysis. See Supplementary methods for more details.

### Omics data preprocessing

For each type of omics analysis, the initial data were represented by a data matrix in which the rows and columns represented the molecules and samples, respectively, and the missing values were set to zero. Then, the data were processed in two steps:

1. Molecules (rows) for which more than half of the samples were zero were removed;

2. For the differential analysis, the remaining rows were normalized as follows:

x_i,j_^'^=(x_i,j_-min_i_)/(max_i_-min_i_)

where *x_i,j_* represents the *j*-th element in row *i* and min*_i_* and max*_i_* represent the minimum and maximum values in row *i*.

For the patient clustering analysis, the remaining rows were standardized by another equation:

Z(x_i,j_)=(x_i,j_-m_i_)/sd_i_

where *x_i,j_* represents the *j*-th element in row *I* and *m_i_* and *sd_i_* represent the mean value and standard deviation of row *i*.

All three sets of omics data were preprocessed with the above processes.

### Differential analysis of the multi-omics data

For the analyses of the preprocessed omics data, when comparing a single disease group and the healthy group, we utilized the Kruskal-Wallis rank sum test to examine the differences, and the fold changes (FCs) were calculated by dividing the mean value of the disease group by the mean value of the healthy group. Molecules with p values less than 0.05 and absolute values of log2 (FC) larger than 1 were recognized as DEMs.

### First step clustering

a) Determination of the clinical factor-relevant elements in the omics data

According to suggestions from physicians experienced in metabolic disease treatment, 8 key clinical factors for 4 basic metabolic diseases were considered; BMI and WaistCir for OB, FPG and OGTT 2hPG for HG, SBP and DBP for HT, and TG and HDL for HP. These clinical indexes were collected. For each type of omics analysis, the Spearman correlation coefficients between each molecule and every clinical index were calculated. Then, the molecules in the omics analyses were ranked based on their absolute correlations with each of the disease-relevant clinical factors (each disease was evaluated with two clinical factors), and the mean absolute correlations were calculated. Finally, the molecules ranked among the top 30% for both clinical factors were selected, and if the number of molecules was larger than 50, we only retained the top 50 based on the mean absolute correlation. This process was repeated for each type of omics analysis, and the results were merged.

b) Clustering based on the clinical factor-relevant elements

The preprocessed data matrixes of the omics datasets were merged, and rows included metabolites, proteins and peptides. Then, the merged matrix was further reduced by only retaining the clinical factor-relevant items. With this matrix (termed *M_r_*), we utilized a consensus clustering strategy to perform the unsupervised clustering of the clinical samples, for which the maximum number of clusters was set to 10, the final cluster number was set to 7, the inner clustering algorithm used was hierarchical clustering and the sample distance was defined as the “1-Pearson correlation”. The clustering method was carried out with the R package ConsensusClusterPlus 45.

### Second step clustering

After the initial clustering analysis, the patients were separated into different clusters. Each cluster was further described according to the mean values of the collected clinical factors, such as LDL, FPG, and ApoB. Then, these clinical factors were ranked by their SDs, and only the top 50% ranked factors were retained. Based on the mean levels of these retained clinical factors for every cluster, we utilized hierarchical clustering (the default clustering method in the R package complexHeatmap 46) to cluster these initial clusters.

### Recognition of the potential clinical and molecular determinants of the identified groups

We evaluated the clinical and molecular differences between each individual group and the combined groups based on the Wilcox test, and the corresponding FC values were computed by dividing the mean value of each group by the mean value of the other groups.

### Random forest-based importance score

Based on the clustering results, we estimated the importance of different molecules and pathways to separate the patients into the identified groups based on a random forest algorithm. This algorithm assesses the feature importance based on the impurity reduction caused by removing the feature from the forest. This was carried out with the R package randomForest 47.

### Evaluation about the performance of molecular features in predicting the redefined three groups

Support vector machine (SVM) algorithm was applied to train the group classifier based on the expression profiles of molecules which showed significant differences between different groups. To evaluate the classifier's performance, we utilized a leave-one-out validation strategy where one individual sample was left out as a testing sample, and the others were taken as training samples, then a SVM classifier was trained and tested based on the training and testing samples respectively, and this processes was repeated until each sample was utilized as a testing sample at once. After the leave-one-out validation, we merged the predicted results of each individual sample, calculated the corresponding specificities and sensitivities, and drawn the ROC curve. The SVM algorithm and ROC curve were respectively carried out based the R package caret 48 and pROC 49.

### Recognition of the heterogeneous molecular network

Based on the three omics datasets and the clinical factor-relevant molecules, we calculated the biweight midcorrelations between any two heterogeneous molecules (a metabolite and a protein, a protein and a peptide, or a peptide and a metabolite) and the corresponding Student p-values with the WGCNA package 50 for the G1, G2 and G3 samples, respectively. Two molecules with an absolute value for the correlation coefficient larger than 0.6 and a p-value less than 0.01 in any of the three groups were included in the heterogeneous molecular network. The resulting network was plotted with Cytoscape 51.

### Pathway enrichment analysis of the molecular network

Pathway information was obtained from the KEGG 52. For each pathway, we determined the metabolites and proteins/genes in the pathway and utilized Fisher's exact test to examine the overlap between the pathway metabolites and the metabolites of interest, as well as the overlap between the pathway proteins/genes (proteins were represented by the corresponding encoding genes) and the proteins of interest.

### GSEA-based pathway activity score

For every individual sample, we generated a ranked molecular list based on the expression profile within one omics dataset. For the metabolomics and proteomics analyses, we could generate the metabolite and protein lists, respectively. However, peptides are not annotated in the KEGG pathways, and we utilized the source proteins of the peptides instead of generating ranked molecular lists in terms of peptidomics. Then, the GSEA method was applied to examine whether the members of a particular pathway were enriched at the top or bottom of the ranked molecular list for the sample, and a GSEA-based pathway activity score was calculated for each pathway as: *GS* =* -log10(p)* if the pathway is up-regulated, and *GS = log10(p)* if the pathway is down-regulated, where *p* is the statistical P-value got from GSEA*.* The GSEA method was performed with the R package piano 53.

### Statistics

All statistical tests and other computations were performed in R. All codes are available upon request. The detailed statistical methods are described in the corresponding sections.

## Figures and Tables

**Table 1:** Demographic characteristics of the collected samples (n=49)

Character	Mean (Standard deviation)
Age (year)	55.60(5.77)
Gender	
Female	19(61%)
Male	30(39%)
Weight (kg)	67.29 (11.05)
Height (cm)	162.49 (6.91)
BMI (kg/m^2^)	25.46 (3.61)
WaistCir (cm)	84.37 (9.93)
FPG (mmol/L)	6.26 (1.68)
OGTT 2hPG (mmol/L)	8.59 (4.31)
HDL (mmol/L)	1.32 (0.32)
LDL (mmol/L)	2.72 (0.76)
TG (mmol/L)	1.32 (0.93)
SBP (mmHg)	128.65 (12.72)
DBP (mmHg)	80.98 (5.89)

2hPG: 2-hour postprandial plasma glucose; BMI: body mass index; DBP: diastolic blood pressure; FPG: fasting plasma glucose; HDL: high density lipoprotein; LDL: low density lipoprotein; OGTT: oral glucose tolerance test; SBP: systolic blood pressure; TG: triglyceride; WaistCir: waist circumference.

**Table 2:** Disease grouping criteria

Disease type	Criteria
Obesity	WaistCir ≥ 85 cm for female and WaistCir ≥ 90 cm for male
Hyperglycemia	FPG ≥ 6.1 mmol/L or OGTT 2hPG ≥ 7.8 mmol/L and/or confirmed diabetes that is under treatment
Hypertension	SBP ≥ 130 mmHg or DBP ≥ 85 mmHg and/or diagnosed and on antihypertensive therapy
Hyperlipidemia	Fasting TG > 1.7 mmol/L or Fasting HDL < 1.04 mmol/L
MTS	Simultaneously meet three or more of the above criteria
T2D	FPG ≥ 7 mmol/L, OGTT 2hPG ≥ 11.1 mmol/L

2hPG: 2-hour postprandial plasma glucose; BMI: body mass index; DBP: diastolic blood pressure; FPG: fasting plasma glucose; HDL: high density lipoprotein; LDL: low density lipoprotein; MTS: metabolic syndrome; OGTT: oral glucose tolerance test; SBP: systolic blood pressure; T2D: type 2 diabetes; TG: triglyceride; WaistCir: waist circumference.

## Abbreviations

0.5hPG: 0.5-hour postprandial plasma glucose
0.5hPSI: 0.5-hour postprandial serum insulin
2hPG: 2-hour postprandial blood glucose
AFAM: afamin
AGC: automatic gain control
ALBU: serum albumin
APOA1: apolipoprotein a-i
ApoB: apolipoprotein b
APOC2: apolipoprotein C-ii
ATP: adult treatment program
AUC: area under curve
%B: pancreatic islet b cell function index
BMI: body mass index
C1: cluster 1
C2: cluster 2
C3: cluster 3
C4: cluster 4
C5: cluster 5
C6: cluster 6
C7: cluster 7
CADH5: cadherin-5
CAH1: carbonic anhydrase 1
CBG: corticosteroid-binding globulin
Cer: ceramide
CFAD: complement factor d
CFAB: complement factor b
CO3: complement factor c3
CO7: complement component c7
CXCL7: c-x-c motif chemokine 7
DBP: diastolic blood pressure
DEM: differentially expressed molecule
DG: diacylglycerol
FC: fold change
FDR: false discovery rate
FFA: free fatty acids
FIBA: fibrinogen alpha chain
FPG: fasting plasma glucose
GPT: glutamic pyruvic transaminase
GOT: glutamic oxalacetic transaminase
GSEA: gene set enrichment analysis
HDL: high density lipoprotein
HEP2: Heparin cofactor 2
HOMA: homoeostasis model assessment
IGKC: immunoglobulin kappa constant
IR: insulin resistance
ITIH4: inter-alpha-trypsin inhibitor heavy chain h4
KEGG: kyoto encyclopedia of genes and genomes
KNG1: kininogen-1
KV113: immunoglobulin kappa variable 1-13
LCAT: lecithin cholesterol acyltransferase
LDL: low density lipoprotein
MS: mass spectrometer
MTS: metabolic syndrome
MYEF2: myelin expression factor 2
OGTT: oral glucose tolerance test
PC: phosphatidylcholine
PD: proteome discoverer
PS: phosphatidylserine
ROC: receiver operating characteristic
%S: insulin sensitivity index
SBP: systolic blood pressure
SRGN: serglycin
SVM: support vector machine
T2D: type 2 diabetes
TBA: total bile acid
T-Bil: total bilirubin
TCH: total cholesterol
TG: triglyceride
THRB: thrombin
TP: total protein
TRFE: serotransferrin
WaistCir: waist circumference
