# Supplementary material for: A multi-omics investigation of the molecular characteristics and classification of six metabolic syndrome relevant diseases
Source: Theranostics. 2020 Jan 12;10(5):2029–46. doi: 10.7150/thno.41106 (PMC7019171; doi:10.7150/thno.41106)
Supplement: Supplementary file 1 — Supplementary figures, tables, and methods. [file thnov10p2029s1.pdf]

Supplementary tables

Table S1. Shared differentially expressed molecules across multiple diseases. Only molecules with literature supports to be associated with multiple diseases were listed.

Type	Name	Summary	Relevant diseases
**Protein**	AFAM	Highly expressed in group b,c,d,e,g	MTS [1, 2]
	MASP1	Highly expressed in group b, d, f	Prediabetes [3], Obesity, Hyperlipidemia [4]
**Metabolites**	glycine	Lowly expressed in group b,c,d,f	T2D [5], obesity [6], MTS [7], Hyperlipidemia [8]
	SM(d16:0/20:0)+H	Highly expressed in group c, f, g	Insulin resistance, MTS [9]
	Cer	Highly expressed in group cand f	Insulin resistance, diabetes [10, 11]
	FFA FFA 18:4	Highly expressed in group c, d, g	Insulin resistance, MTS [12]
**Source proteins of the polypeptides**	ALBU	Highly expressed in group c,d,f,g	MTS [13], T2D [14, 15], Obesity [16], Hyperlipidemia [17]
	ITIH4	Lowly expressed in group c, e, f	Metabolically healthy obesity [18, 19]
	APOA1	Lowly expressed in group b, c, f	MTS [20], T2D[21], Hyperlipidemia

Table S2. Disease specific differentially expressed molecules. Only molecules with literature supports to be associated with the specific disease were listed.

Disease	Molecular type	Molecule	References
**Obesity**	Protein	CAMP	[22]
		IGLC2 /IGLC6	[23]
	Metabolite	epinephrine	[24]
		C0−carnitine	[25]
		Hypoxanthine	[26]
		L−proline	[27]
		Pyruvic acid	[28]
	Polypeptide source protein	IGHG1	[23]
		FETUA	[29]
		FIBA	[30]
**MTS**	Protein	APOC2	[31]
		RET4	[32]
	Metabolites	TG	
		DG	[33]
		FFA	[12]
		CDCA	[34, 35]
	Polypeptide source protein	FIBA	[36]
		CO3	[37]
		A1AT	[38]
**Hyperglycemia**	Protein	CADH5	[39]
		CBG	[40]
		FIBA	[41, 42]
		GPV	[43]
	Metabolites	Ethanolamine	[44]
		Serine	[45]
	Polypeptide source protein	FIBA	[41, 42]
		TRFE	[46]
		CO3	[47]
		IGKC	[48]
**Hypertension**	Metabolites	Carnitine	[49]
		Indoxyl sulfate	[50]
	Polypeptide source protein	FIBA	[51, 52]
		ALBU	[53-55]
**Hyperlipidemia**	Protein	LBP	[56, 57]
		FIBA	[58]
		CO3	[59]
**T2D**	Protein	CAH1	[60]
	Metabolites	PC(20:1/22:6)+H	[61]
		KNG1	[62]
		MYEF2	[63]

Table S3. The peptides corresponding to the abbreviated peptide names in Figure 6.

Node name	Peptide
A1AT-pp1	EDPQGDAAQKTD
A1AT-pp2	EDPQGDAAQKTDTS
A1AT-pp3	EDPQGDAAQKTDTSHHD
A1AT-pp4	EDPQGDAAQKTDTSHHDQD
AACT-pp1	HPNSPLDEENLTQENQD
ALBU-pp1	DAHKSEVAHRF
ALBU-pp2	DAHKSEVAHRFKDLG
ALBU-pp3	HKSEVAHRFKDLG
AMBP-pp3	GDEELLRFS
APOA1-pp1	DEPPQSPWD
APOA1-pp2	EEYTKKLNTQ
APOA1-pp3	LSALEEYTKKLNTQ
APOL1-pp1	EEAGARVQQNVPSGTD
APOL1-pp2	EEAGARVQQNVPSGTDTGD
CO3-pp1	SEETKENEGFTVTAEGK
CXCL7-pp1	NLAKGKEESLDSD
FETUA-pp1	APHGPGLIYRQPN
FIBA-pp1	ADSGEGDFLAEGGGV
FIBA-pp2	FTSSTSYNRGDST
FIBA-pp3	FTSSTSYNRGDSTFESKSYKMA
FIBA-pp4	KMADEAGSEADHEGTHST
FIBA-pp5	NRGDSTFESKSY
FIBA-pp6	SSYSKQFTSSTSYNRGDSTFESKS
FIBA-pp7	STSYNRGDSTFES
FIBA-pp8	SSYSKQFTSSTSYNRGDST
HEMO-pp1	TPLPPTSAHGNVAEGETKPD
HEP2-pp1	GSKGPLDQLEKGGETAQSAD
IGHG1-pp1	EALHNHYTQKSLSLSPG
IGHG1-pp2	ALHNHYTQKSLSLSPG
IGKC-pp1	NALQSGNSQESVTEQD
IGKC-pp2	NALQSGNSQESVTEQDSKD
IGLC3-pp1	SSPVKAGVETTTPSKQ
ITIH4-pp1	NVHSGSTF
K1C9-pp1	SRSGGGGGGGLGSGGSIRSSY
KV105-pp1	DIQMTQSPSTLSASVGD
KV116-pp1	DIQMTQSPSSLSASVGD
THRB-pp1	QVTVAMTPR
THRB-pp2	GLDEDSDRAIEGR
TRFE-pp1	VAFVKHQTVPQNTGGKNPD

Supplementary Figures

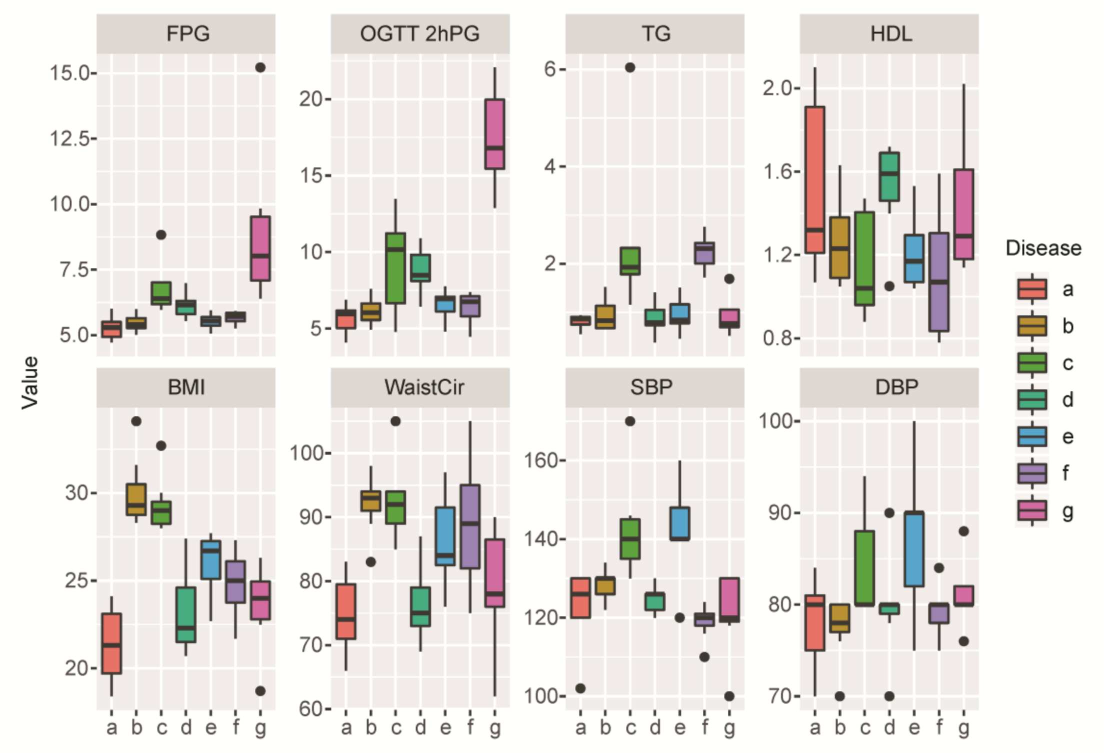

Figure S1 Boxplots of clinical differences between all collected seven groups of samples. The centers of the boxes represent the median values. The bottom and top boundaries of the boxes represent the 25th and 75th percentiles. The whiskers indicate 1.5 times of the interquartile range. The dots represent points falling outside this range. 2hPG: 2-hour postprandial plasma glucose; BMI: body mass index; DBP: diastolic blood pressure; FPG: fasting plasma glucose; HDL: high density lipoprotein; OGTT: oral glucose tolerance test; SBP: systolic blood pressure; TG: triglyceride; WaistCir: waist circumference.

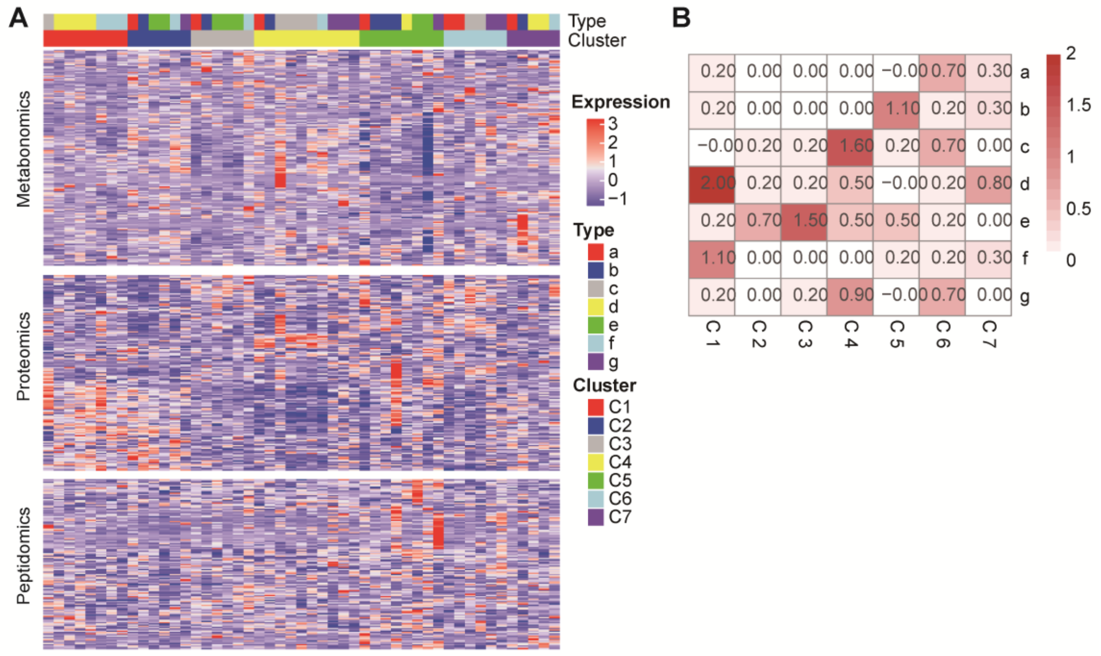

Figure S2 Clustering the samples based only on the omics data. **A** Clustering results. The central heatmap displays the standardized expression of metabolomics, proteomics and peptidomics. Each column corresponds to a patient and each row corresponds to a molecule. The top two rows indicate the original patient groups (a to g), the clustering results (C1 to C7). **B** Significance of the overlaps between the different clusters (represented by the columns) and the patient groups (represented by the rows) according to Fisher's exact test.

Supplementary methods

Metabolome profiling

Metabolomics and lipidomics profiling was performed with a Waters UPLC system coupled with a Q Exactive HF mass spectrometer (Thermo Fisher Scientific, Rockford, IL, U.S.A.) [64, 65]. The separation was performed with a 2.1×100 mm ACQUITY^{TM} 1.7 μm C8 column in ESI positive ion mode, and the mobile phase consisted of water with 0.1% formic acid (A) and acetonitrile (B). The gradient program was as follows: 5% B, held for 1 min, linearly changed to 100% B within 24 min, held for 4 min, then returned to 5% B, held 2 min. For the ESI negative ion mode, the separation was performed with a 2.1×100 mm ACQUITY^{TM} 1.8 μm T3 column, and the mobile phase consisted of 6.5 mM ammonium bicarbonate water solution (C) and 6.5 mM ammonium bicarbonate in 95% methanol and water (D). The gradient program was as follows: 2% D, held for 1 min, linearly changed to 100% D over 18 min, held for 4 min, then returned to 2% D, held for 2 min. The flow rate was 0.35 mL/min, and the column temperature was kept at 50 ℃ in positive ion mode and 55 ℃ in negative ion mode.

The separation of the lipid metabolites was performed with a Waters UPLC C8 ACQUITY column (2.1 mm × 100 mm × 1.7 μm) (Milford, MA, USA). The elution solvent consisted of A (ACN:H2O = 60:40, v/v) and B (IPA:ACN = 90:10, v/v), both of which contained 10 mM ammonium acetate. The elution program started at 32% B for the initial 1.5 min, followed by a linear increase to 85% B over the next 14 min. Over 0.1 min, solvent B was rapidly increased to 97% and then maintained for 2.4 min for a column flush. Subsequently, the elution solvent was returned to 32% B within 0.1 min and maintained for 2 min for column equilibration. The column temperature was set to 55 ℃, and the flow rate was 0.26 ml/min. The spray voltage was 3.5 kV for positive mode and 3.0 kV for negative mode. The capillary temperature was maintained at 300 ℃. The auxiliary gas heater temperature was 350 ℃. The flow rates of the sheath gas and the auxiliary gas were 45 arb and 10 arb, respectively. The S-lens RF level was 50. The AGC target was set to a 3106 ion capacity, and the maximum IT was 200 ms. The mass resolution was set to 120,000 and 30,000 for full scan MS and data-dependent MS/MS, respectively.

GC-MS analysis was also performed for the metabolic profiling. A QP 2010 GC-MS system (Shimadzu, Japan) with a DB-5 MS fused silica capillary column (30 m × 0.25 mm × 0.25 μm, Agilent Technologies, USA) was used. A pseudotargeted GC-MS metabolomics method was used as previously reported [66].

Quality control (QC) samples were prepared by mixing equal aliquots of serum from each real sample, and QC samples were run (one QC after each 8 serum samples). The reproducibility of the metabolite ions was also evaluated with relative standard deviation (RSD%) in the QC samples. In this study, 78.3% of ions had RSD% less than 20%, and 91.1% of ions had RSD% less than 30%.

Proteome profiling

One microliter of serum was diluted with 100 μL 25 mM ammonia bicarbonate (ABC, pH 8.5), and the protein concentration was determined by a BCA assay (Beyotime, China). The proteins were reduced in 20 mM dithiothreitol (DTT, Merck, Germany) at 95 ℃ for 5 min, and the products were alkylated in 40 mM iodoacetamide (IAA, Merck, Germany) at room temperature in the dark for 30 min. Next, 2 μg of trypsin (Promega, WI) was added and incubated at 37 ℃ for 16 h.

Each sample was analyzed in technical triplicate with a nano-RPLC-MS/MS on a Q-Exactive MS (Thermo, CA) coupled with an Easynano LC system (Thermo, CA). The mobile phases were buffer A (2% acetonitrile, 98% water, and 0.1% formic acid) and B (98% acetonitrile, 2% water, and 0.1% formic acid). Fused silica capillaries were purchased from Sino Sumtech (Handan, China). A C18 trap column (150 μm i.d. × 5 cm) was connected to a capillary separation column (75 μm i.d. × 15 cm). Both the trap and separation columns were made in-house and packed with Daiso C18 particles (5 μm, 100 Å, Osaka, Japan). A 110 min gradient was established, comprising 60 min of 6%−22% buffer B, followed by 30 min of 22%−35% buffer B, 10 min of 35%−80% buffer B and 10 min of 80% buffer B. The spray voltage was 2.5 kV, and the temperature of the ion transfer capillary was set to 275 ℃. The Q Exactive MS was operated in positive ion mode, and the 10 most intense ions were subjected to HCD fragmentation with a normalized collision energy of 28%. The MS scans were performed at a resolution of 70 000 from m/z 300 to 1800 (automatic gain control (AGC) value, 1E6; maximum injection time, 100 ms), and the data were acquired in profile mode. The MS/MS scans were performed at a resolution of 17 500 (AGC, 1E5; maximum injection time, 60 ms), and the data were acquired in centroid mode using a 20 s exclusion window. The unassigned ions or those with a charge of +1 and >+7 were rejected. Then, the raw data were uploaded into Maxquant (v.1.6.1.0) and searched against the UniProtKB human complete proteome sequence database (release 2017_06, 24,148 entries). The search included cysteine carbamidomethylation as the fixed modification and methionine oxidation and acetylation of protein N-terminal as variable modifications. The searching tolerance for precursor ions was 10 ppm, and that for fragment ions was 20 ppm. Matching between runs with retention time window of 0.7 min and the label free quantification algorithm were performed. During proteome profiling, each individual sample was measured three times, and the average profiling result was adopted as the final one. The samples were profiled in two batches, and batch effects were removed with the R package sva [67].

Peptidome profiling

A total of 50 μL of serum supernatant was diluted with 250 μL of deionized water and denatured by boiling for 5 min. Then, the mixture was transferred to a centrifugal ultrafiltration tube and centrifuged (Eppendorf Centrifuge 5804 R) at 6000 g for 40 min. After washing twice with deionized water and 20% acetonitrile solution (v/v) containing 0.1% formic acid (v/v), all of the filtrate was collected, freeze-dried and redissolved in 50 μL of 0.1% formic acid solution (v/v). Twenty-five microliters of each diluted filtrate was mixed together to generate each QC sample. The remaining individual sample and QC sample were used for light and heavy labeling via a stable isotope dimethyl labeling method [68].

The peptide analysis was performed with nano-RPLC-ESI-MS/MS on an LTQ-Orbitrap Elite mass spectrometer coupled with a Dionex UltiMate 3000 RSLC-nano System (Thermo, San Jose, CA). The samples were loaded onto a 3 cm C18 trap column (200 μm i.d.) at a flow rate of 3 μL/min and separated on a 15 cm C18 column (150 μm i.d.) at a flow rate of 500 nL/min. The mobile phases A (100% water and 0.1% formic acid) and B (80% acetonitrile, 20% water, and 0.1% formic acid) were used with a 105 min gradient: 2% B for 10 min, 2–5% B for 3 min, 5–28% B for 60 min, 28–45% B for 15 min, 45–95% B for 1 min and 95% B for 5 min. The LTQ-Orbitrap Elite mass spectrometer was operated in a positive, data-dependent MS/MS acquisition mode. The ion transfer capillary temperature was 275 ℃, and the spray voltage was 2.7 kV. A full mass scan was acquired with the Orbitrap mass

analyzer from m/z 350 to 1650 at a resolution of 120000, and the top 20 parent ions with charge states ≥ 2 in the full scan were fragmented by collision-induced dissociation (CID) with 35% normalized collision energy. The dynamic exclusion function parameters were as follows: repeat count 1, repeat duration 30 s, and exclusion duration 90 s. The acquired raw MS/MS spectra from each sample were searched against the International Protein Index (IPI) human database with the UniProt website using Mascot Version 2.4.1 (Matrix Science). MaxQuant software (version 1.6) was used to perform the quantitative analysis. The search parameters for both searching modes were as follows: no specific proteolytic enzyme was specified; the oxidation of methionine (M) was the variable modification; the false discovery rate (FDR) cutoff was 0.01. Additionally, the precursor-ion mass error tolerance was 20 ppm, and the fragment-ion mass error tolerance was 0.8 Da for the Mascot search. Duplicates with dimethyl Lys 0 and N-term 0 as the light labels and dimethyl Lys 6 and N-term 6 as the heavy labels were selected for the Maxquant search.

Supplementary References

1. Kronenberg F, Kollerits B, Kiechl S, Lamina C, Kedenko L, Meisinger C, et al. Plasma concentrations of afamin are associated with the prevalence and development of metabolic syndrome. Circ Cardiovasc Genet. 2014;7:822-9.
2. Seeber B, Morandell E, Lunger F, Wildt L, Dieplinger H. Afamin serum concentrations are associated with insulin resistance and metabolic syndrome in polycystic ovary syndrome. Reprod Biol Endocrinol. 2014;12:88.
3. von Toerne C, Huth C, de Las Heras Gala T, Kronenberg F, Herder C, Koenig W, et al. MASP1, THBS1, GPLD1 and ApoA-IV are novel biomarkers associated with prediabetes: the KORA F4 study. Diabetologia. 2016;59:1882-92.
4. Frauenknecht V, Thiel S, Storm L, Meier N, Arnold M, Schmid JP, et al. Plasma levels of mannan-binding lectin (MBL)-associated serine proteases (MASPs) and MBL-associated protein in cardio- and cerebrovascular diseases. Clin Exp Immunol. 2013;173:112-20.
5. De Luca G, Calpona PR, Caponetti A, Macaione V, Di Benedetto A, Cucinotta D, et al. Preliminary report: amino acid profile in platelets of diabetic patients. Metabolism. 2001;50:739-41.
6. Oberbach A, Bluher M, Wirth H, Till H, Kovacs P, Kullnick Y, et al. Combined proteomic and metabolomic profiling of serum reveals association of the complement system with obesity and identifies novel markers of body fat mass changes. J Proteome Res. 2011;10:4769-88.
7. Badoud F, Lam KP, DiBattista A, Perreault M, Zulyniak MA, Cattrysse B, et al. Serum and adipose tissue amino acid homeostasis in the metabolically healthy obese. J Proteome Res. 2014;13:3455-66.
8. Yamakado M, Nagao K, Imaizumi A, Tani M, Toda A, Tanaka T, et al. Plasma Free Amino Acid Profiles Predict Four-Year Risk of Developing Diabetes, Metabolic syndrome, dyslipidemia, and hypertension in Japanese Population. Sci Rep. 2015;5:11918.
9. Brice SE, Cowart LA. Sphingolipid metabolism and analysis in metabolic disease.

Adv Exp Med Biol. 2011;721:1-17.
10. Chavez JA, Summers SA. A ceramide-centric view of insulin resistance. Cell Metab. 2012;15:585-94.
11. Chaurasia B, Summers SA. Ceramides - Lipotoxic Inducers of Metabolic Disorders. Trends Endocrinol Metab. 2015;26:538-50.
12. Ormseth MJ, Swift LL, Fazio S, Linton MF, Raggi P, Solus JF, et al. Free fatty acids are associated with metabolic syndrome and insulin resistance but not inflammation in systemic lupus erythematosus. Lupus. 2013;22:26-33.
13. Cho HM, Kim HC, Lee JM, Oh SM, Choi DP, Suh I. The association between serum albumin levels and metabolic syndrome in a rural population of Korea. J Prev Med Public Health. 2012; 45: 98-104.
14. Schmidt MI, Duncan BB, Sharrett AR, Lindberg G, Savage PJ, Offenbacher S, et al. Markers of inflammation and prediction of diabetes mellitus in adults (atherosclerosis risk in communities study): a cohort study. Lancet. 1999;353:1649-52.
15. Kunutsor SK, Khan H, Laukkanen JA. Serum albumin concentration and incident type 2 diabetes risk: new findings from a population-based cohort study. Diabetologia. 2015;58:961-7.
16. Liu M, Tang J, Zeng J, He Y. Higher serum albumin was related with diabetes incidence and the impact of BMI changes: based on cohort study of 18,384 Chinese male elderly. J Diabetes Complications. 2017;31:1663-8.
17. Akboga MK, Yayla C, Balci KG, Ozeke O, Maden O, Kisacik H, et al. Relationship between serum albumin level and monocyte-to-high-density lipoprotein cholesterol ratio with saphenous vein graft disease in coronary bypass. Thorac Cardiovasc Surg. 2017;65:315-21.
18. Doumatey AP, Zhou J, Zhou M, Prieto D, Rotimi CN, Adeyemo A. proinflammatory and lipid biomarkers mediate metabolically healthy obesity: A proteomics study. Obesity. 2016;24:1257-65.
19. Wen W, Zheng W, Okada Y, Takeuchi F, Tabara Y, Hwang JY, et al. Meta-analysis of genome-wide association studies in East Asian-ancestry populations identifies four new loci for body mass index. Hum Mol Genet. 2014;23:5492-504.
20. Zhong L, Li Q, Jiang Y, Cheng D, Liu Z, Wang B, et al. The ApoB/ApoA1 ratio is associated with metabolic syndrome and its components in a Chinese population. Inflammation. 2010;33(6):353-8.
21. Mooradian AD, Haas MJ, Wong NC. Transcriptional control of apolipoprotein A-I gene expression in diabetes. Diabetes. 2004;53:513-20.
22. Hoang-Yen Tran D, Hoang-Ngoc Tran D, Mattai SA, Sallam T, Ortiz C, Lee EC, et al. Cathelicidin suppresses lipid accumulation and hepatic steatosis by inhibition of the CD36 receptor. Int J Obes (Lond). 2016;40:1424-34.
23. Marzullo P, Minocci A, Giarda P, Marconi C, Tagliaferri A, Walker GE, et al. Lymphocytes and immunoglobulin patterns across the threshold of severe obesity. Endocrine. 2014;45:392-400.
24. Ziegler MG, Milic M, Sun P, Tang CM, Elayan H, Bao X, et al. Endogenous epinephrine protects against obesity induced insulin resistance. Auton Neurosci.

2011;162:32-4.
25. Cha YS. Effects of L-carnitine on obesity, diabetes, and as an ergogenic aid. Asia Pac J Clin Nutr. 2008;17:306-8.
26. Saiki S, Sato T, Kohzuki M, Kamimoto M, Yosida T. Changes in serum hypoxanthine levels by exercise in obese subjects. Metabolism. 2001;50:627-30.
27. Bagheri M, Djazayery A, Farzadfar F, Qi L, Yekaninejad MS, Aslibekyan S, et al. Plasma metabolomic profiling of amino acids and polar lipids in Iranian obese adults. Lipids Health Dis. 2019;18:94.
28. Pennington AW. Pyruvic acid metabolism in obesity. Am J Dig Dis. 1955;22:33-7.
29. Andersen G, Burgdorf KS, Sparsø T, Borch-Johnsen K, Jørgensen T, Hansen T, et al. AHSGTag single nucleotide polymorphisms associate with type 2 diabetes and dyslipidemia. Diabetes. 2008;57:1427-32.
30. Hafez M, El-Masry S, Musa N, Fathy M, Hassan M, Hassan N, et al. Relationship between visceral obesity and plasma fibrinogen in obese children. J Pediatr Endocr Met. 2016;29:289-96.
31. Fallaize R, Carvalho-Wells AL, Tierney AC, Marin C, Kiec-Wilk B, Dembinska-Kiec A, et al. APOE genotype influences insulin resistance, apolipoprotein CII and CIII according to plasma fatty acid profile in the Metabolic Syndrome. Sci Rep. 2017;7:6274.
32. Yang Q, Graham TE, Mody N, Preitner F, Peroni OD, Zabolotny JM, et al. Serum retinol binding protein 4 contributes to insulin resistance in obesity and type 2 diabetes. Nature. 2005;436:356-62.
33. Polewski MA, Burhans MS, Zhao M, Colman RJ, Shanmuganayagam D, Lindstrom MJ, et al. Plasma diacylglycerol composition is a biomarker of metabolic syndrome onset in rhesus monkeys. J Lipid Res. 2015;56:1461-70.
34. Hansen M, Scheltema MJ, Sonne DP, Hansen JS, Sperling M, Rehfeld JF, et al. Effect of chenodeoxycholic acid and the bile acid sequestrant colesevelam on glucagon-like peptide-1 secretion. Diabetes Obes Metab. 2016;18:571-80.
35. Ethanic M, Stanimirov B, Pavlovic N, Golocorbin-Kon S, Al-Salami H, Stankov K, et al. Pharmacological applications of bile acids and their derivatives in the treatment of metabolic syndrome. Front Pharmacol. 2018;9:1382.
36. Imperatore G, Riccardi G, Iovine C, Rivellese AA, Vaccaro O. Plasma fibrinogen: a new factor of the metabolic syndrome: a population-based study. Diabetes Care. 1998;21:649-54.
37. Xin Y, Hertle E, van der Kallen CJH, Schalkwijk CG, Stehouwer CDA, van Greevenbroek MMJ. Complement C3 and C4, but not their regulators or activated products, are associated with incident metabolic syndrome: the CODAM study. Endocrine. 2018;62:617-27.
38. Setoh K, Terao C, Muro S, Kawaguchi T, Tabara Y, Takahashi M, et al. Three missense variants of metabolic syndrome-related genes are associated with alpha-1 antitrypsin levels. Nat Commun. 2015;6:7754.
39. Bernard S, Loffroy R, Sérusclat A, Boussel L, Bonnefoy E, Thévenon C, et al. Increased levels of endothelial microparticles CD144 (VE-Cadherin) positives in type

2 diabetic patients with coronary noncalcified plaques evaluated by multidetector computed tomography (MDCT). Atherosclerosis. 2009;203:429-35.
40. Fernandez-Real J-M, Pugeat M, Grasa M, Broch M, Vendrell J, Brun J, et al. Serum corticosteroid-binding globulin concentration and insulin resistance syndrome: a population study. J Clin Endocrinol Metab. 2002;87:4686-90.
41. Bembde AS. A study of plasma fibrinogen level in type-2 diabetes mellitus and its relation to glycemic control. Indian J Hematol Blo. 2012;28:105-8.
42. Jain A, Gupta HL, Narayan S. Hyperfibrinogenemia in patients of diabetes mellitus in relation to glycemic control and urinary albumin excretion rate. J Assoc Physicians India. 2001;49:227-30.
43. Schneider DJ. Factors contributing to increased platelet reactivity in people with diabetes. Diabetes Care. 2009;32:525-7.
44. Tao H, Zhang Y, Zeng X, Shulman GI, Jin S. Niclosamide ethanolamine-induced mild mitochondrial uncoupling improves diabetic symptoms in mice. Nat Med. 2014;20:1263-9.
45. Holm LJ, Haupt-Jorgensen M, Larsen J, Giacobini JD, Bilgin M, Buschard K. L-serine supplementation lowers diabetes incidence and improves blood glucose homeostasis in NOD mice. PloS One. 2018;13:e0194414.
46. Golizeh M, Lee K, Ilchenko S, Ösme A, Bena J, Sadygov RG, et al. Increased serotransferrin and ceruloplasmin turnover in diet-controlled patients with type 2 diabetes. Free Radic Biol Med. 2017;113:461-9.
47. Engström G, Hedblad B, Eriksson K-F, Janzon L, Lindgärde F. Complement C3 is a risk factor for the development of diabetes: a population-based cohort study. Diabetes. 2005;54:570-5.
48. Guo X, Meng G, Liu F, Zhang Q, Liu L, Wu H, et al. Serum levels of immunoglobulins in an adult population and their relationship with type 2 diabetes. Diabetes Res Clin Pr. 2016;115:76-82.
49. Mate A, Miguel-Carrasco JL, Vazquez CM. The therapeutic prospects of using L-carnitine to manage hypertension-related organ damage. Drug Discov Today. 2010;15:484-92.
50. Kaminski TW, Pawlak K, Karbowska M, Znorko B, Mor AL, Mysliwiec M, et al. The impact of antihypertensive pharmacotherapy on interplay between protein-bound uremic toxin (indoxyl sulfate) and markers of inflammation in patients with chronic kidney disease. Int Urol Nephrol. 2019;51:491-502.
51. Shankar A, Wang JJ, Rochtchina E, Mitchell P. Positive association between plasma fibrinogen level and incident hypertension among men: population-based cohort study. Hypertension. 2006;48:1043-9.
52. Sonkodi S, Sohar I, Stumpf E. Correlation of the fibrinogen level with blood pressure and plasma renin activity in rats with early Goldblatt hypertension. Acta Physiol Hung. 1987;70:403-7.
53. Oda E. Serum albumin may prevent hypertension by inhibiting angiotensin converting enzyme. Intern Med. 2014;53:2411.
54. Oda E. Decreased serum albumin predicts hypertension in a Japanese health screening population. Intern Med. 2014;53:655-60.

55. Snipelisky D, Jentzer J, Batal O, Dardari Z, Mathier M. Serum albumin concentration as an independent prognostic indicator in patients with pulmonary arterial hypertension. Clin Cardiol. 2018;41:782-7.
56. Tilves CM, Zmuda JM, Kuipers AL, Nestlerode CS, Evans RW, Bunker CH, et al. Association of lipopolysaccharide-binding protein with aging-related adiposity change and prediabetes among African ancestry Men. Diabetes Care. 2016;39:385-91.
57. Liu X, Lu L, Yao P, Ma Y, Wang F, Jin Q, et al. Lipopolysaccharide binding protein, obesity status and incidence of metabolic syndrome: a prospective study among middle-aged and older Chinese. Diabetologia. 2014;57:1834-41.
58. Eliasson M, E Evrin P, Lundblad D. Fibrinogen and fibrinolytic variables in relation to anthropometry, lipids and blood pressure: the northern Sweden MONICA study. J Clin Epidemiol. 1994;47:513-24.
59. Barbu A, Hamad OA, Lind L, Ekdahl KN, Nilsson B. The role of complement factor C3 in lipid metabolism. Molecular Immunology. 2015;67:101-7.
60. Bellei E, Rossi E, Lucchi L, Uggeri S, Albertazzi A, Tomasi A, et al. Proteomic analysis of early urinary biomarkers of renal changes in type 2 diabetic patients. Proteom Clin Appl. 2008;2:478-91.
61. Li Y, Wang DD, Chiuve SE, Manson JE, Willett WC, Hu FB, et al. Dietary phosphatidylcholine intake and type 2 diabetes in men and women. Diabetes Care. 2015;38:e13-4.
62. Merchant ML, Niewczas MA, Ficociello LH, Lukenbill JA, Wilkey DW, Li M, et al. Plasma kininogen and kininogen fragments are biomarkers of progressive renal decline in type 1 diabetes. Kidney Int. 2013;83:1177-84.
63. Rachana KS, Manu MS, Advirao GM. Insulin influenced expression of myelin proteins in diabetic peripheral neuropathy. Neurosci Lett. 2016;629:110-5.
64. Zhao X, Zeng Z, Chen A, Lu X, Zhao C, Hu C, et al. Comprehensive strategy to construct in-house database for accurate and batch identification of small molecular metabolites. Anal Chem. 2018;90:7635-43.
65. Liu X, Li J, Zheng P, Zhao X, Zhou C, Hu C, et al. Plasma lipidomics reveals potential lipid markers of major depressive disorder. Anal Bioanal Chem. 2016;408:6497-507.
66. Zhou Y, Hu C, Zhao X, Luo P, Lu J, Li Q, et al. Serum metabolomics study of gliclazide-modified-release-treated type 2 diabetes mellitus patients using a gas chromatography-mass spectrometry method. J Proteome Res. 2018;17:1575-85.
67. Leek JT, Johnson WE, Parker HS, Jaffe AE, Storey JD. The sva package for removing batch effects and other unwanted variation in high-throughput experiments. Bioinformatics. 2012;28:882-3.
68. Boersema PJ, Raijmakers R, Lemeer S, Mohammed S, Heck AJ. Multiplex peptide stable isotope dimethyl labeling for quantitative proteomics. Nat Protoc. 2009;4:484-94.
